# Early-life tobacco exposure is causally implicated in aberrant RAG-mediated recombination in childhood acute lymphoblastic leukemia

**DOI:** 10.1038/s41375-024-02407-3

**Published:** 2024-09-09

**Authors:** Tanxin Liu, Keren Xu, Anmol Pardeshi, Swe Swe Myint, Alice Y. Kang, Libby M. Morimoto, Michael R. Lieber, Joseph L. Wiemels, Scott C. Kogan, Catherine Metayer, Adam J. de Smith

**Affiliations:** 1 Center for Genetic Epidemiology, Department of Population and Public Health Sciences, University of Southern California Keck School of Medicine, Los Angeles, CA USA; 2 USC Norris Comprehensive Cancer Center, University of Southern California, Los Angeles, CA USA; 3 School of Public Health, University of California Berkeley, Berkeley, CA USA; 4 Departments of Pathology, Biochemistry, Molecular Microbiology & Immunology, and Section of Molecular & Computational Biology (Department of Biological Sciences), University of Southern California, Los Angeles, CA USA; 5 Department of Laboratory Medicine, Helen Diller Family Comprehensive Cancer Center, University of California San Francisco, San Francisco, CA USA

**Keywords:** Acute lymphocytic leukaemia, Cancer epidemiology, Cancer genetics, Cancer genomics, Risk factors

The development of childhood acute lymphoblastic leukemia (ALL) typically involves formation of preleukemic clones in early-life followed by the postnatal acquisition of “second-hit” mutations and copy-number alterations that drive progression to overt leukemia [1]. “Off-target” V(D)J recombination is a mechanism known to drive the formation of deletions in ALL [2, 3]. The recombination-activating gene (RAG) proteins, encoded by RAG1 and RAG2, typically help to generate antibody diversity by inducing DNA double-strand breaks and recombining the variable (V), diversity (D), and joining (J) gene segments during the early stage of B-cell and T-cell maturation, resulting in diverse immunoglobulins and T-cell receptors (Ig/TCR) [1, 4].

We previously found the frequency of driver gene deletions in childhood ALL patients was positively associated with early-life tobacco smoke exposure [5, 6]. Our prior studies were limited to analysis of eight genes commonly deleted in ALL and targeted by a multiplex ligation-dependent probe amplification assay, which did not resolve breakpoint sequences [6]. Previous studies demonstrating that increased off-target deletions mediated by V(D)J recombination were associated with passive maternal tobacco exposure [7] as well as hematologic malignancies [3] motivated us to investigate whether tobacco exposure during pregnancy may be associated with RAG recombination-mediated deletions in childhood ALL. Here, we performed whole genome sequencing (WGS) in childhood ALL patients with high or low prenatal tobacco smoke exposure, extending upon our prior investigations by examining structural variation genome-wide and mutational mechanisms.

Childhood ALL patients were included from the California Childhood Leukemia Study (CCLS), described in **Supplementary Methods** and in detail elsewhere [8]. ALL patients were categorized as having “high” (*N* = 18) or “low” (*N* = 17) early-life tobacco exposure based on established epigenetic biomarkers (Fig. S1, see **Supplementary Methods**) [5, 6, 9]. Paired tumor-normal WGS was performed for the 35 patients, and quality control assessment, methods for detecting somatic variants (including single nucleotide variants [SNVs], indels, and structural variants [SVs]), mutational signature analyses, and all statistical tests are described in **Supplementary Methods**. Two-sided *p*-values < 0.05 were considered statistically significant.

The majority of patients (31/35) were of the B-cell immunophenotype. Patient demographic data are included in Tables S1 and S2. Patients harbored a median of 1729 SNVs and 535 indels, with affected ALL driver genes including *KRAS*, *FLT3*, *JAK2*, *PAX5*, *ERG, PTPN11, NF1*, and *RB1* (Fig. S2). The number of SNVs (*p* = 0.198) or indels (*p* = 0.843) were not significantly different between high and low tobacco exposure patients (Fig. 1). We identified a median of 33 SVs, 12 deletions, 2 duplications, 6 inversions, and 4 translocations per patient, with a median of 6 SVs overlapping known ALL driver genes, including *CDKN2A/B*, *IKZF1*, *VPREB1*, and *P2RY8* (Fig. S3). Expanding upon our previous finding for 8 ALL driver genes [5, 6], we found a significantly higher number of deletions genome-wide (*p* = 0.001), as well as a higher frequency of translocations (*p* = 0.002), inversions (*p* = 0.004), duplications (*p* = 0.017), and overall SVs (*p* < 0.001), in the high tobacco exposure patients compared with low exposure patients (Fig. 1, Table S1). This suggests that early-life tobacco smoke exposure may be associated with general genomic instability in ALL tumor samples, or perhaps that tobacco exposure persists throughout childhood in high-exposure patients. We note that when limiting to SVs overlapping ALL driver genes, only total SVs and deletions remained significantly increased in the high-exposure group (Table S3, Fig. S4); other types of SV may be passenger events associated with tobacco exposure or potentially confounded by molecular subtype. Age-at-diagnosis was positively associated with number of SVs (*p* = 0.009) and deletions (*p* = 0.0005) (Table S4).

**Fig. 1:**
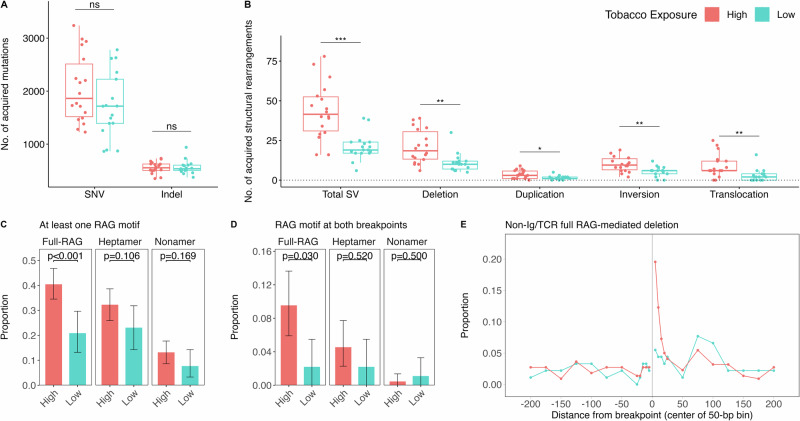
Somatic alterations and RAG-mediated deletions by prenatal tobacco smoke exposure status in childhood ALL patients. Analysis of somatic alterations was conducted using whole-genome sequencing data in 35 paired tumor-normal samples. The number of different somatic alteration types in the high tobacco exposure (*n* = 18) and low tobacco exposure (*n* = 17) childhood ALL patients is displayed by box and whisker plots for (**A**) single nucleotide variants (SNVs) and insertion deletion polymorphisms (indels), and (**B**) structural variants (SVs: deletions, duplications, inversions, translocations). Statistical comparisons were performed using Wilcoxon rank sum tests (***P < 0.001; **P < 0.01; *P < 0.05). Analysis of off-target (non-Ig/TCR) RAG-mediated deletions in the high and low tobacco exposure patients was performed using FIMO. Bar plots display the proportion of deletions in non-Ig/TCR regions with at least one breakpoint (**C**) or with both breakpoints (**D**) having an RSS motif in childhood ALL patients with high (*n* = 18) or low (*n* = 17) tobacco exposure. A total of 220 non-Ig/TCR region deletions were detected in high tobacco exposure patients and 91 non-Ig/TCR deletions in low exposure patients. Error bars represent 95% bootstrapped confidence intervals. Chi-square tests were used to compare the proportions of deletions with at least one RAG motif (either full, heptamer or nonamer) between two groups. Fisher’s exact tests were used to compare the proportions of deletions with a RAG motif at both breakpoints between two groups. **E** The proportion of non-Ig/TCR (*i.e*., off-target) putatively RAG-mediated deletions with at least one full RSS motif was plotted against the distance of the motif from the deletion breakpoint, ranging from within 5-bp to 200-bp. A positive distance represents bases interior to the deletion breakpoint (inside the deletion) and a negative value represents bases exterior to the breakpoint (outside the deletion). Proportions are displayed for the high (*n* = 18) and low (*n* = 17) tobacco exposure patients separately.

To explore the hypothesis that tobacco exposure-related gene deletions in childhood ALL are RAG-mediated, we searched deletion breakpoint sequences for occurrence of RAG motifs using FIMO [10] (details in **Supplementary Methods**). We considered presence of the full recombination signal sequence (RSS) motif at one or both breakpoints as strong evidence of RAG recombination (Fig. S5) and presence of only heptamer or nonamer motifs as weaker evidence. Among 566 total deletions, 255 (45.1%) had at least one breakpoint located in Ig/TCR regions (on-target), and 311 (54.9%) deletions had both breakpoints in non-Ig/TCR regions and may therefore be mediated by off-target RAG recombination. Ninety-three percent of Ig/TCR deletions and 35% of non-Ig/TCR deletions had a full RSS motif within 50 bp of at least one of two breakpoints (*i.e*., putatively RAG-mediated). High tobacco exposure patients had a higher total number of putatively RAG-mediated deletions than the low exposure group overall (*p* = 0.002), and when limited to non-Ig/TCR deletions (*p* = 0.003) or Ig/TCR deletions (*p* = 0.005). High tobacco exposure was also associated with a significantly higher number of deletions with a full RSS motif at both breakpoints in non-Ig/TCR regions (*p* = 0.004) but not in Ig/TCR regions (*p* = 0.472) (Table S5).

As the number of RAG-mediated deletions in the high versus low tobacco exposure patients may reflect the frequency of overall deletions in each patient group, we next examined the *proportion* of deletions that appeared to be mediated by off-target RAG recombination. In non-Ig/TCR regions, high-exposure patients harbored a significantly higher proportion of putatively RAG-mediated deletions than low-exposure patients (40.5% vs. 20.9%; *p* = 0.001) (Fig. 1C, Table S6, Fig. S6). We also identified a higher proportion of non-Ig/TCR deletions with the full RSS motif at both breakpoints in high versus low exposure patients (9.5% vs 2.2%; *p* = 0.0297) (Fig. 1D). This off-target effect was in the opposite direction of the on-target effects observed in Ig/TCR regions (Fig. S7), suggesting a potential skewing towards off-target RAG recombination in the high tobacco exposure group.

In support that these are true RAG-mediated events, the RSS motif (full RSS, heptamer or nonamer) was largely internal to deletion breakpoints in both patient groups (Fig. 1E, Fig. S8A, B). Further, of heptamers located internal to deletion breakpoints, 121/124 (97.6%) at non-Ig/TCR deletions and 394/398 (99.0%) at Ig/TCR deletions were found in the correct orientation for typical V(D)J recombination where the RAG motifs are deleted in the form of “excision circles”. Analysis of non-templated nucleotide sequences at deletion breakpoints provided additional support for RAG-mediated deletions, as described in **Supplementary Methods** and **Results**.

De novo motif analysis using HOMER (see **Supplementary Methods**) identified the RAG heptamer as significantly enriched at deletion breakpoints (Fig. S9). Consistent with FIMO results, high tobacco exposure patients harbored a higher proportion of deletions with at least one off-target RAG heptamer (high vs. low groups: 32.7% vs 18.7%; *p* = 0.013) and a higher proportion of deletions with off-target RAG heptamer at both breakpoints (6.4% vs 1.1%; *p* = 0.076) (Fig. S10, Table S7). No additional significant motifs identified by HOMER had a target frequency above 5% (Fig. S11).

Patient age-at-diagnosis was positively associated with both the number (*p* = 0.0007) and proportion (*p* = 0.005) of non-Ig/TCR putatively RAG-mediated deletions (Table S4). Patient age may be a proxy for cumulative dose of tobacco exposure, there may be age-related differences in molecular subtypes that vary by RAG-mediated deletions, or a longer latency period between exposure and ALL diagnosis may provide more time for somatic alterations to develop.

In a multilevel model, which accounted for the varying number of deletions in each patient, non-Ig/TCR deletions identified among the high tobacco exposure patients had 2.44-fold higher odds (95% CI:1.13–5.38) of being putatively RAG-mediated than deletions in the low exposure group (Table 1), with an even stronger association when the full RSS motif was found at both breakpoints (OR = 4.70, 95% CI:1.34–29.75). Further analyses and statistical modeling of putatively RAG-mediated deletions, including in relation to age-at-diagnosis, ethnicity, and additional features, are presented in Supplementary Information and Table S8.

**Table 1:** Multilevel model analysis of prenatal tobacco smoke exposure and off-target RAG-mediated deletions in childhood ALL patients.

Full RSS motif at deletion breakpoints*	Univariable model**			Multivariable model**	
	Tobacco exposure, OR (95% CI)	Age-at-diagnosis, OR (95% CI)	Hispanic/Latino ethnicity***, OR (95% CI)	Tobacco exposure, adjusting for age-at-diagnosis, OR (95% CI)	Tobacco exposure, adjusting for age-at-diagnosis and ethnicity, OR (95% CI)
One or both breakpoints	2.44 (1.13, 5.38)	1.12 (1.02, 1.24)	1.22 (0.56, 2.75)	1.95 (0.88, 4.41)	2.17 (0.95, 5.13)
Both breakpoints	4.70 (1.34, 29.75)	1.06 (0.96, 1.23)	1.47 (0.61, 3.90)	4.41 (1.16, 28.96)	5.81 (1.42, 39.84)

*FIMO was used to identify a full RSS motif (heptamer and nonamer motif plus 12- or 23-nucleotide spacer) within 50 bp of one or both deletion breakpoints.

**Multilevel logistic regression models were used to estimate the association between prenatal tobacco exposure (18 high exposure vs. 17 low exposure patients) and having a full RSS motif at one or both breakpoints of non-Ig-TCR (i.e., “off-target”) deletions (*n* = 311).

***Analyses were limited to self-reported Hispanic/Latino vs. non-Hispanic due to sample size.

Finally, we explored whether any mutational signatures were associated with tobacco exposure but found no significant differences between high and low exposure patients (details in **Supplementary Results**, Table S9–14, Figs. S12–16).

Altogether, our results support a potential leukemogenic role of early-life tobacco exposure, a preventable environmental factor, in childhood ALL development. Case-control studies of parental smoking based on questionnaire data, which are subject to misclassification bias, have shown inconsistent associations with childhood ALL risk [11–13]. We recently reported a lack of association between DNA methylation at the *AHRR* CpG cg05575921, an epigenetic biomarker of maternal smoking during pregnancy, and childhood ALL risk [14], which supported previous evidence regarding all childhood ALL combined. Although results from our case-only analyses appear inconsistent with case-control study findings, they suggest that tobacco smoke exposure may have tumor subtype-specific effects on ALL development.

There is a paucity of evidence on the potential influence of environmental factors on RAG recombination activity, although tobacco smoke exposure was previously implicated in off-target RAG recombination in cord blood lymphocytes [7, 15]. The role of early-life tobacco exposure in childhood ALL may not necessarily be directly mutagenic but instead have effects on the developing immune system, for example through upregulation of RAG proteins or via stalling of lymphocyte development given that RAG proteins are most active in immature lymphocytes, which warrants future investigation.

Our study has some limitations. Small sample size limited statistical power and our ability to adjust for potential confounders, such as molecular subtype. Tobacco exposure was analyzed as a binary variable, and potential dose-response relationships were not examined. Epigenetic biomarkers of prenatal tobacco exposure were derived from newborn dried bloodspots, although it is possible that these may be correlated with postnatal exposure to parental smoking during childhood, a more relevant time window of exposure given that second-hit deletions in ALL typically arise postnatally [1]. We also cannot rule out other unmeasured environmental exposures that may correlate with prenatal tobacco exposure. Further studies are needed to confirm our findings and to understand the precise biological mechanisms and timing of exposures that underlie the association between tobacco exposure and deletions in childhood ALL.

## Supplementary information

Supplementary Information

Supplementary Tables

Supplementary Datasets

## Data Availability

This study used biospecimens from the California Biobank Program. Any uploading of genomic data and/or sharing of these biospecimens or individual data derived from these biospecimens has been determined to violate the statutory scheme of the California Health and Safety Code Sects. 124980(j), 124991(b), (g), (h), and 103850 (a) and (d), which protect the confidential nature of biospecimens and individual data derived from biospecimens. Should we be contacted regarding individual-level data contributing to the findings reported in this study, inquiries will be directed to the California Department of Public Health Institutional Review Board to establish an approved protocol to utilize the data, which cannot otherwise be shared peer-to-peer. Full results for somatic mutations and structural variants identified in each tumor sample by whole-genome sequencing and results of RSS motif analysis are included in the **Supplementary Data** files.

## References

[1] Greaves M. A causal mechanism for childhood acute lymphoblastic leukaemia. Nat Rev Cancer. 2018;18:471–84. 29784935 10.1038/s41568-018-0015-6 PMC6986894

[2] Mullighan CG, Miller CB, Radtke I, Phillips LA, Dalton J, Ma J, et al. BCR–ABL1 lymphoblastic leukaemia is characterized by the deletion of Ikaros. Nature. 2008;453:110–4. 18408710 10.1038/nature06866

[3] Papaemmanuil E, Rapado I, Li Y, Potter NE, Wedge DC, Tubio J, et al. RAG-mediated recombination is the predominant driver of oncogenic rearrangement in ETV6-RUNX1 acute lymphoblastic leukemia. Nat Genet. 2014;46:116–25. 24413735 10.1038/ng.2874 PMC3960636

[4] Park S-R. Activation-induced cytidine deaminase in B cell immunity and cancers. Immune Netw. 2012;12:230–9. 23396757 10.4110/in.2012.12.6.230 PMC3566417

[5] Xu K, Li S, Whitehead TP, Pandey P, Kang AY, Morimoto LM, et al. Epigenetic biomarkers of prenatal tobacco smoke exposure are associated with gene deletions in childhood acute lymphoblastic leukemia. Cancer Epidemiol Biomark Prev. 2021;30:1517–25. 10.1158/1055-9965.EPI-21-0009 PMC8338876 34020997

[6] De Smith AJ, Kaur M, Gonseth S, Endicott A, Selvin S, Zhang L, et al. Correlates of prenatal and early-life tobacco smoke exposure and frequency of common gene deletions in childhood acute lymphoblastic leukemia. Cancer Res. 2017;77:1674–83. 28202519 10.1158/0008-5472.CAN-16-2571 PMC5380517

[7] Finette BA, O’Neill JP, Vacek PM, Albertini RJ. Gene mutations with characteristic deletions in cord blood T lymphocytes associated with passive maternal exposure to tobacco smoke. Nat Med. 1998;4:1144–51. 9771747 10.1038/2640

[8] Metayer C, Zhang L, Wiemels JL, Bartley K, Schiffman J, Ma X, et al. Tobacco smoke exposure and the risk of childhood acute lymphoblastic and myeloid leukemias by cytogenetic subtype. Cancer Epidemiol Biomark Prev. 2013;22:1600–11. 10.1158/1055-9965.EPI-13-0350 PMC3769478 23853208

[9] Reese SE, Zhao S, Wu MC, Joubert BR, Parr CL, Håberg SE, et al. DNA methylation score as a biomarker in newborns for sustained maternal smoking during pregnancy. Environ Health Perspect. 2017;125:760–6. 27323799 10.1289/EHP333 PMC5381987

[10] Grant CE, Bailey TL, Noble WS. FIMO: scanning for occurrences of a given motif. Bioinformatics. 2011;27:1017–8. 21330290 10.1093/bioinformatics/btr064 PMC3065696

[11] Stjernfeldt M, Berglund K, Lindsten J, Ludvigsson J. Maternal smoking during pregnancy and risk of childhood cancer. Lancet. 1986;1:1350–2. 2872471 10.1016/s0140-6736(86)91664-8

[12] Orsi L, Rudant J, Ajrouche R, Leverger G, Baruchel A, Nelken B, et al. Parental smoking, maternal alcohol, coffee and tea consumption during pregnancy, and childhood acute leukemia: the ESTELLE study. Cancer Causes Control. 2015;26:1003–17. 25956268 10.1007/s10552-015-0593-5

[13] Milne E, Greenop KR, Scott RJ, Bailey HD, Attia J, Dalla-Pozza L, et al. Parental prenatal smoking and risk of childhood acute lymphoblastic leukemia. Am J Epidemiol. 2012;175:43–53. 22143821 10.1093/aje/kwr275

[14] Zhong C, Li S, Arroyo K, Morimoto LM, de Smith AJ, Metayer C, et al. Gene-environment analyses reveal novel genetic candidates with prenatal tobacco exposure in relation to risk for childhood acute lymphoblastic leukemia. Cancer Epidemiol Biomark Prev. 2023;32:1707–15. 10.1158/1055-9965.EPI-23-0258 PMC11812055 37773025

[15] Keohavong P, Xi L, Day RD, Zhang L, Grant SG, Day BW, et al. HPRT gene alterations in umbilical cord blood T-lymphocytes in newborns of mothers exposed to tobacco smoke during pregnancy. Mutat Res. 2005;572:156–66. 15790499 10.1016/j.mrfmmm.2005.01.014

