# Supplementary material for: Early-life tobacco exposure is causally implicated in aberrant RAG-mediated recombination in childhood acute lymphoblastic leukemia
Source: Leukemia. 2024 Sep 9;38(11):2492–6. doi: 10.1038/s41375-024-02407-3 (PMC11518992; doi:10.1038/s41375-024-02407-3)
Supplement: Supplementary file 1 — Supplementary Information [file 41375_2024_2407_MOESM1_ESM.pdf]

Supplementary Information

Early-life tobacco exposure is causally implicated in aberrant RAG-mediated recombination in childhood acute lymphoblastic leukemia

Supplementary Methods	pg. 2-9
Supplementary Results	pg. 10-12
Supplementary References	pg. 13-17
Supplementary Figures	pg. 18-34

Supplementary Methods

Selection of ALL patients

This study was reviewed and approved by the Institutional Review Boards at the University of Southern California, the University of California, Berkeley, the California Department of Public Health, and all participating hospitals. Written informed consent was obtained from all study participants. This study was conducted in accordance with the Declaration of Helsinki.

Childhood ALL patients were included from the California Childhood Leukemia Study (CCLS), a case-control study conducted from 1995 to 2015 to identify genetic and environmental risk factors for childhood leukemia. Briefly, ALL patients were identified within 72 hours after diagnosis at hospitals and were eligible for the enrollment if they: (i) were younger than 15 years of age-at-diagnosis, (ii) had no previous cancer diagnosis, (iii) were diagnosed at one of the participating hospitals, (iv) lived in California for at least three months, and (v) had an English or Spanish-speaking parent or guardian. Children of all self-reported race/ethnicities were eligible.

We previously determined DNA methylation levels at the *AHRR* CpG cg05575921 and calculated epigenetic smoking scores in 478 childhood ALL patients, including 194 assayed with the Illumina HumanMethylation450K BeadChip® arrays, and 284 assayed with the Illumina EPIC methylation array as previously described [1-2]. For the current study, we selected two groups of ALL patients that we categorized as having "high" or "low" early-life tobacco smoke exposure, based on the combined results from two established epigenetic biomarkers: CpG cg05575921 in the *AHRR* gene, and an epigenetic smoking score consisting of up to 28 CpGs [1]. Both biomarkers have been

strongly associated with prenatal tobacco smoke exposure in newborn blood samples. In brief, the *AHRR* CpG cg05575921 was the top most statistically significant CpG related to maternal smoking during pregnancy [2–5], and the DNA methylation-based smoking score was derived from a linear combination of 28 previously selected maternal smoking-associated CpGs [1].

To identify the high or low tobacco exposure patients for WGS, we first ranked subjects based on each epigenetic biomarker, separately in the 450K and EPIC array datasets given the potential differences in DNA methylation for cg05575921 and for additional CpGs included in the epigenetic score (**Figure S1**). For *AHRR* CpG cg05575921, at which DNA methylation has an inverse relationship with tobacco exposure, individuals with the lowest to highest DNA methylation were ranked from highest to lowest. For the epigenetic score, the individual with the highest score was given the highest ranking. Next, we summed the ranks across the two biomarkers for an overall ranking for each subject. We aimed to select 20 ALL cases with high tobacco exposure and 20 cases with low exposure based on their overall rank score and the availability of both tumor and germline DNA for WGS. Due to sample availability, we ultimately included 18 ALL cases in the high tobacco exposure group and 17 cases in the low exposure group in our WGS analyses (**Figure S1**).

Whole-genome sequencing

DNA was isolated from diagnostic bone marrow (“tumor”) samples using the Qiagen DNA Blood Mini Kit and from newborn dried blood spot specimens using the Qiagen QIAamp DNA Investigator Kit, which provided median yields of 612 and 348 ng DNA, respectively.

WGS was performed by Novogene for the 35 matched tumor-normal pairs with Illumina Novaseq 6000 technology, using 150 bp paired-end libraries and obtaining an average coverage of ~35X coverage for germline samples and 58X coverage for tumor samples. We performed data processing and quality control of WGS data using the Genome Analysis Tool Kit (GATK) release 4.2.6.1 following best practices guidelines [6,7].

Quality control assessment of raw sequencing data was performed using FastQC [8], and reads were mapped to Human Reference genome version 38 (GRCh38) using the BWA-MEM 0.7.17 [9]. Duplicate reads were marked using Picard v2.27.1 MarkDuplicates. Base quality scores were recalibrated using GATK ApplyBQSR, with known sites including dbSNP138, 1000 genome phase 1 SNPs, Mills and 1000G gold standard indels, and Homo Sapiens assembly 38 known indels from the GATK resource bundle.

Somatic variant calling

Somatic single nucleotide variants (SNVs) and indels were called from 35 matched tumor-normal pairs using GATK Mutect2 [10] in tumor-normal mode, with a panel of normal callset that was created from 40 publicly available normal samples from the 1000 Genome project to capture common recurring artifacts. The Genome Aggregation Database (gnomAD) [11] VCF was used as a reference for germline population allele frequencies to measure the likelihood that a variant call in the normal might be a germline variant instead of an artifact. Raw SNVs and indels were filtered based on the probability of somatic variants using FilterMutectCalls after calculating the cross-sample contamination estimates and learning orientation bias artifacts. To exclude false-positive calls, variants

that were marked as "PASS" in the previous step were filtered using the following criteria: coverage depth ≥ 14X for tumor samples and ≥ 10X for paired normal samples, and variant allele fraction (VAF) ≥ 0.10 [12]. We further restricted analyses to autosomal chromosomes in the downstream analyses. Variants were annotated using the Ensembl Variant Effect Predictor (VEP) v110.0 and ANNOVAR v2019-10-24 [13].

Identification of structural variants

We identified structural variants (SV) including deletions, duplications, inversions and translocations using three complementary tools: Manta v1.6.0 [14], Lumpy v0.2.14 [15], and Delly v1.1.6 [16] (**Figure S17**). Lumpy was run using the wrapper Smoove v.0.2.3. SV calls were merged into a union set using the SURVIVOR tool, retaining only those called by at least two methods with the maximum allowed distance of 100bp as measured pairwise between breakpoints (begin 1 vs. begin 2, end1 vs. end 2) [17]. Variants were removed if they: 1) did not pass Delly, Manta or Lumpy, or passed only one of the three callers; 2) had a VAF <0.10; or 3) were 50bp or smaller in size. SVs were annotated using AnnotSV 3.2.2 with two annotation modes: one directly related to the full-length SV (full mode) and the other related to each gene within SV (split mode) [18]. Known ALL driver genes were identified based on the previous literature [19–22].

Deletion breakpoint motif analysis

We obtained +/- 50bp flanking sequences from each deletion breakpoint based on hg38 coordinates. Recombination signal sequence (RSS) motif enrichment analysis was performed using the Find Individual Motif Occurrences (FIMO) tool in MEME suite v5.5.5

($P<10^{-4}$) [23,24]. In brief, FIMO searches a set of individual sequences for the occurrence of known motifs provided by the user, treating each motif independently [23]. The position-weight matrix (PWM) used to identify RSS motifs were obtained from previous studies [25–27], assuming a background rate of 0.2 for C/G and 0.3 for A/T. We investigated the presence of the full RSS motif (**Figure S5**), which can include a 12- or 23-nucleotide spacer, and heptamer and nonamer motifs within 50 bp flanking each deletion breakpoint. This was conducted initially for deletions in both immunoglobulin/T-cell receptor (Ig/TCR) and non-Ig/TCR regions, and subsequently limited to non-Ig/TCR regions (where both breakpoints were outside +/- 1000 bp of Ig/TCR regions) to examine off-target RAG recombination. The coordinates of "on-target" Ig/TCR (IgH, IgK, IgL, TRB, TRA/TRD, TRG) regions were based on prior studies [19,28] (**Table S15**). To explore the distance and clustering of motifs, we identified the motif signal decay within 5-200 bp from deletion breakpoints and plotted the proportion of deletions with at least one RSS motif.

De novo deletion breakpoint motif analysis was conducted using HOMER v.4.11 [29]. We selected +/- 50bp from the deletion breakpoints and used repeat masked sequences. We first searched for motifs ranging from 5 to 12 bp and then specified the length of motifs to be 7 bp (heptamer). We did not investigate the full RSS motif using HOMER as the recommended maximum motif length was 15 bp.

Analysis of non-templated nucleotides

Non-templated nucleotides (NTN) inserted at deletion breakpoints, a hallmark of RAG recombination [26], were identified by comparing the prefix of the upstream longest right soft-clipped sequence with the suffix of the downstream longest left soft-clipped sequence

and finding the longest common sequence. Upstream and downstream 50-bp sequences from the deletion breakpoints were extracted from BAM files using pysam v.0.22.0, a wrapper of samtools [30]. For deletions with microhomology at the breakpoints, to avoid false positive NTN calls we checked whether the first few base pairs of the prefix of the downstream right matched sequence (of the read with left soft-clipped sequence) was the same as for the suffix of the upstream left matched sequence (of the read with right soft-clipped sequence) and also was the same as the previously mentioned longest common sequence. We also manually checked 20 deletions using the Integrative Genomics Viewer (IGV) v2.13.0 to confirm our findings: of 20 non-Ig/TCR deletions with RAG motif enrichment at both breakpoints, manual inspection using IGV revealed that 19 (95%) deletions had NTN, similar to the number (n=18, 90%) identified by the overlapped segment between the upstream and downstream deleted sequences.

Mutational signature analysis

Tobacco-associated mutagenesis involves both endogenous and exogenous mutational processes, with each process producing distinct mutation characteristics referred to as mutational signatures [31,32]. Two distinct approaches were used for analyzing mutational signatures. First, we conducted de novo extraction of mutational signatures using SigProfilerExtractor v1.1.4.[33]. Extracted signatures were decomposed into the set of reference signatures from Catalogue of Somatic Mutations in Cancer (COSMIC) database [34]. The optimal set of de novo signatures were extracted by the nonnegative matrix factorization algorithm and were matched to a set of reference signatures from COSMIC database.

Second, we assigned previously known (reference) mutational signatures to the mutational profile of each individual sample using SigProfilerAssignment v0.0.33 [35] and deconstructSigs v1.9.0 [36]. SigprofilerAssignment supports the probabilistic assignment of known mutational signatures to each individual sample [35]. Different from de novo extraction, fitting known signatures to each mutation within each sample can be used for small cohorts and clinical evaluation for individual patients. DeconstructSigs was used to replicate the results [36].

Statistical analysis

All analyses were performed using *R* v4.3.3. Demographic and tumor characteristics were compared between high and low tobacco exposure groups by Wilcoxon rank-sum test for continuous variables, or by Chi-square test or Fisher's exact test for categorical variables. Two-sample Wilcoxon rank-sum tests were used to compare the frequency of different mutation events (including number of SNVs, indels, deletions, duplications, inversions, and translocations), number or proportion of mutations assigned to each mutational signature, and number of RAG-mediated deletions between high and low tobacco exposure groups. Chi-square test was used to compare the proportion of putatively RAG-mediated deletions between each group. Fisher's exact test was used to compare the proportion of deletions with RSS motif at both breakpoints, and the proportion of patients identified with each mutational signature between the two groups, as some events were less than five. Multilevel logistic regression models with random intercept were used to estimate the association between prenatal tobacco exposure and the likelihood of a deletion having an RSS motif near at least one breakpoint or at both breakpoints,

adjusting for patient race/ethnicity and age-at-diagnosis. Univariable linear regression models and the Spearman correlation coefficient tests were used to assess the association between age-at-diagnosis and number of SNVs, indels, SVs and mutational signatures. Two-sided $p < 0.05$ was considered statistically significant.

In multilevel logistic regression models, each deletion was coded as 1 or 0 based on the presence or absence of RSS motifs at the breakpoints. The number of clusters corresponded to the number of patients (n=35), and the number of observations corresponded to the number of deletions. To scrutinize the above association, we conducted the following sensitivity analyses: 1) removing large deletions (>1Mb); 2) excluding four *ETV6*::*RUNX1* cases that are known to harbor RAG-mediated deletions; 3) excluding 6 deletions where RSS motifs were only found external to the breakpoints and were, thus, less likely to be RAG-mediated [27].

Supplementary Results

Non-templated nucleotide (NTN) sequences at deletion breakpoints

Among 311 non-Ig/TCR deletions, 231 (74.3%) had NTN at deletion breakpoints: the proportion was highest for those with the RSS motif at both breakpoints (92.31%), followed by those with the RSS motif at only one breakpoint (79.3%), and then non-RAG-mediated deletions (64.53%). These results were similar to those previously reported in ALL patients by Papaemmanuil et al., in which NTN sequences were found at 84.0% of RAG-mediated deletions with resolved breakpoints but only 65.3% of non-RAG-mediated deletions [26].

Multilevel model analysis

Including age-at-diagnosis and self-reported Hispanic/Latino ethnicity in a multilevel model attenuated the strength of the association between high prenatal tobacco exposure and the odds of non-Ig/TCR deletions being putatively RAG-mediated (OR: 2.17 [95%CI: 0.95, 5.13] vs. OR:2.44 [95%CI: 1.13, 5.38] in the model without age-at-diagnosis and ethnicity). We repeated our analysis focusing on deletions for which the full RSS motif was found at both breakpoints and found that high tobacco exposure was strongly associated with RAG recombination in both univariable (OR, 4.70, 95%CI: 1.34, 29.75) and multivariable (OR, 5.81, 95%CI: 1.42, 39.84) models (**Table 1**).

Deletions associated with off-target RAG recombination in ALL patients have been previously described as being on average smaller than non-RAG-mediated deletions; for example, in Papaemmanuil et al. [26], 97.5% (116/119) of likely RAG-mediated deletions were <1Mb in size compared with only 78.6% (77/98) of non-RAG-mediated deletions.

Similarly, in our data, 107/108 (99.7%) of putatively RAG-mediated non-Ig/TCR deletions were <1Mb in size. Thus, we repeated the multilevel model analysis restricting to 274 non-Ig/TCR deletions that were <1Mb in size. This revealed a stronger association between tobacco exposure and putatively RAG-mediated deletions in the univariable (OR: 2.55, 95%CI: 1.23, 5.32) and multivariable (OR: 2.16; 95%CI: 0.98, 4.82) models (**Table S8**).

Four of the high tobacco exposure patients were identified as harboring the *ETV6*::*RUNX1* fusion in our SV analysis, whereas no *ETV6*::*RUNX1* fusions were found in the low exposure group. Excluding the 4 *ETV6*::*RUNX1* patients, the association retained significance in the univariable model (OR: 2.35, 95% CI: 1.02, 5.56) and exhibited a suggestive trend in the fully-adjusted model (OR: 1.85, 95% CI: 0.71, 4.89). Furthermore, after removing 6 deletions which only had RSS motifs external to breakpoints, we observed stronger associations in the univariable (OR, 2.57, 95%CI:1.17, 5.78) and fully-adjusted models (OR, 2.30, 95%CI: 0.98, 5.60) (**Table S8**).

Mutational signature analysis

In the *de novo* signature analysis using SigProfilerExtractor, four *de novo* SBS were considered as the best solution and were decomposed into seven COSMIC signatures: SBS1 and SBS5 (clock-like), SBS2 and SBS13 (AID/APOBEC activity), SBS3 (defective homologous recombination-based DNA damage repair), SBS18 (possibly damage by reactive oxygen species (ROS)), and SBS30 (deficiency in base excision repair due to inactivating mutations in *NTHL1*) (**Table S9-10, Figure S12-S13**). The AID/APOBEC signature was identified in only two of the 35 patients, both of which were in the high-

tobacco exposure group. For signatures detected in both tobacco exposure groups (clock-like and ROS), we did not observe any significant difference in either total number of signature-related mutations between exposure groups or proportion of signature-related mutations in each patient between the two groups (*p*>0.05) (**Table S10-11**, **Figure S13**). No significant difference was found for the proportion of subjects carrying each of the signatures between the two groups (**Table S12**). Age-at-diagnosis was positively associated with the number of SBS1-related mutations (linear regression beta:30.89, *p*=0.0003). Similar to SBS results, we found no significant difference between high and low tobacco exposure patients for any indel or double-base substitution signatures (**Table S9**).

A total of 18 SBS signatures were identified using SigProfilerAssignment, including the above 7 signatures and an additional 11 signatures: SBS7a, SBS7b, SBS8, SBS9, SBS40, SBS44, SBS37, SBS39, SBS89, SBS54 and SBS58 (**Figure S14**). SBS2/SBS13, SBS3 and SBS44 were only observed in the high tobacco exposure group. Again, there was no significant difference in the total number or proportion of mutations assigned to each signature between two tobacco exposure groups (**Table S13-14, Figure S15**). Age-at-diagnosis was positively associated with the number of SBS1-related mutations (beta:28.07, *p*=0.0005).

DeconstructSigs results identified a total of 13 SBS signatures: SBS1, SBS2, SBS3, SBSB5, SBS7, SBS8, SBS9, SBS12, SBS13, SBS16, SBS18, SBS25 and unknown signature (**Figure S16**). Consistent with results from SigProfilerExtractor and SigProfilerAssignment, two subjects with high prenatal tobacco exposure had SBS2 and SBS13.

References

1. Reese SE, Zhao S, Wu MC, Joubert BR, Parr CL, Håberg SE, et al. DNA Methylation Score as a Biomarker in Newborns for Sustained Maternal Smoking during Pregnancy. Environ Health Perspect. 2017;125: 760–766.

2. Xu K, Li S, Whitehead TP, Pandey P, Kang AY, Morimoto LM, et al. Epigenetic Biomarkers of Prenatal Tobacco Smoke Exposure Are Associated with Gene Deletions in Childhood Acute Lymphoblastic Leukemia. Cancer Epidemiol Biomarkers Prev. 2021;30: 1517–1525.

3. De Smith AJ, Kaur M, Gonseth S, Endicott A, Selvin S, Zhang L, et al. Correlates of Prenatal and Early-Life Tobacco Smoke Exposure and Frequency of Common Gene Deletions in Childhood Acute Lymphoblastic Leukemia. Cancer Res. 2017;77: 1674–1683.

4. Gonseth S, de Smith AJ, Roy R, Zhou M, Lee S-T, Shao X, et al. Genetic contribution to variation in DNA methylation at maternal smoking-sensitive loci in exposed neonates. Epigenetics. 2016;11: 664–673.

5. Joubert BR, Felix JF, Yousefi P, Bakulski KM, Just AC, Breton C, et al. DNA Methylation in Newborns and Maternal Smoking in Pregnancy: Genome-wide Consortium Meta-analysis. Am J Hum Genet. 2016;98: 680–696.

6. Van der Auwera GA, Carneiro MO, Hartl C, Poplin R, Del Angel G, Levy-Moonshine A, et al. From FastQ data to high confidence variant calls: the Genome Analysis Toolkit best practices pipeline. Curr Protoc Bioinformatics. 2013;43: 11.10.1-11.10.33.

7. McKenna A, Hanna M, Banks E, Sivachenko A, Cibulskis K, Kernytsky A, et al. The Genome Analysis Toolkit: a MapReduce framework for analyzing next-generation DNA sequencing data. Genome Res. 2010;20: 1297–1303.

8. Andrews, S. et al. FastQC: a quality control tool for high throughput sequence data. 2010. Available: http://www.bioinformatics.babraham.ac.uk/projects/fastqc

9. Li H. Aligning sequence reads, clone sequences and assembly contigs with BWA-MEM. arXiv [q-bio.GN]. 2013. Available: http://arxiv.org/abs/1303.3997

10. Benjamin D, Sato T, Cibulskis K, Getz G, Stewart C, Lichtenstein L. Calling Somatic SNVs and Indels with Mutect2. bioRxiv. 2019. p. 861054. doi:10.1101/861054

11. Karczewski KJ, Francioli LC, Tiao G, Cummings BB, Alföldi J, Wang Q, et al. The mutational constraint spectrum quantified from variation in 141,456 humans. Nature. 2020;581: 434–443.

12. Cibulskis K, Lawrence MS, Carter SL, Sivachenko A, Jaffe D, Sougnez C, et al. Sensitive detection of somatic point mutations in impure and heterogeneous cancer samples. Nat Biotechnol. 3/2013;31: 213–219.

13. Wang K, Li M, Hakonarson H. ANNOVAR: functional annotation of genetic variants from high-throughput sequencing data. Nucleic Acids Res. 2010;38: e164.

14. Chen X, Schulz-Trieglaff O, Shaw R, Barnes B, Schlesinger F, Källberg M, et al. Manta: rapid detection of structural variants and indels for germline and cancer sequencing applications. Bioinformatics. 2016;32: 1220–1222.

15. Layer RM, Chiang C, Quinlan AR, Hall IM. LUMPY: a probabilistic framework for structural variant discovery. Genome Biol. 2014;15: R84.

16. Rausch T, Zichner T, Schlattl A, Stütz AM, Benes V, Korbel JO. DELLY: structural variant discovery by integrated paired-end and split-read analysis. Bioinformatics. 2012;28: i333–i339.

17. Jeffares DC, Jolly C, Hoti M, Speed D, Shaw L, Rallis C, et al. Transient structural variations have strong effects on quantitative traits and reproductive isolation in fission yeast. Nat Commun. 2017;8: 14061.

18. Geoffroy V, Guignard T, Kress A, Gaillard J-B, Solli-Nowlan T, Schalk A, et al. AnnotSV and knotAnnotSV: a web server for human structural variations annotations, ranking and analysis. Nucleic Acids Res. 2021;49: W21–W28.

19. Studd JB, Cornish AJ, Hoang PH, Law P, Kinnersley B, Houlston R. Cancer drivers and clonal dynamics in acute lymphoblastic leukaemia subtypes. Blood Cancer J. 2021;11: 177.

20. Roberts KG, Li Y, Payne-Turner D, Harvey RC, Yang Y-L, Pei D, et al. Targetable Kinase-Activating Lesions in Ph-like Acute Lymphoblastic Leukemia. N Engl J Med. 2014;371: 1005–1015.

21. Gu Z, Churchman ML, Roberts KG, Moore I, Zhou X, Nakitandwe J, et al. PAX5-driven subtypes of B-progenitor acute lymphoblastic leukemia. Nat Genet. 2019;51: 296–307.

22. Brady SW, Roberts KG, Gu Z, Shi L, Pounds S, Pei D, et al. The genomic landscape of pediatric acute lymphoblastic leukemia. Nat Genet. 2022;54: 1376–1389.

23. Grant CE, Bailey TL, Noble WS. FIMO: scanning for occurrences of a given motif. Bioinformatics. 2011;27: 1017–1018.

24. Bailey TL, Boden M, Buske FA, Frith M, Grant CE, Clementi L, et al. MEME SUITE: tools for motif discovery and searching. Nucleic Acids Res. 2009;37: W202-8.

25. Machado HE, Mitchell E, Øbro NF, Kübler K, Davies M, Leongamornlert D, et al. Diverse mutational landscapes in human lymphocytes. Nature. 2022;608: 724–732.

26. Papaemmanuil E, Rapado I, Li Y, Potter NE, Wedge DC, Tubio J, et al. RAG-mediated recombination is the predominant driver of oncogenic rearrangement in ETV6-RUNX1 acute lymphoblastic leukemia. Nat Genet. 2/2014;46: 116–125.

27. Hesse JE, Lieber MR, Mizuuchi K, Gellert M. V(D)J recombination: a functional definition of the joining signals. Genes Dev. 1989;3: 1053–1061.

28. Lefranc M-P. Nomenclature of the human T cell receptor genes. Curr Protoc Immunol. 2001;Appendix 1: A.1O.1-A.1O.23.

29. Heinz S, Benner C, Spann N, Bertolino E, Lin YC, Laslo P, et al. Simple Combinations of Lineage-Determining Transcription Factors Prime cis-Regulatory Elements Required for Macrophage and B Cell Identities. Mol Cell. 05/2010;38: 576–589.

30. Li H, Handsaker B, Wysoker A, Fennell T, Ruan J, Homer N, et al. The Sequence Alignment/Map format and SAMtools. Bioinformatics. 2009;25: 2078–2079.

31. Alexandrov LB, Ju YS, Haase K, Van Loo P, Martincorena I, Nik-Zainal S, et al. Mutational signatures associated with tobacco smoking in human cancer. Science. 2016;354: 618–622.

32. Alexandrov LB, Nik-Zainal S, Wedge DC, Campbell PJ, Stratton MR. Deciphering Signatures of Mutational Processes Operative in Human Cancer. Cell Rep. 01/2013;3: 246–259.

33. Islam SMA, Díaz-Gay M, Wu Y, Barnes M, Vangara R, Bergstrom EN, et al. Uncovering novel mutational signatures by de novo extraction with SigProfilerExtractor. Cell Genom. 2022;2: None.

34. Tate JG, Bamford S, Jubb HC, Sondka Z, Beare DM, Bindal N, et al. COSMIC: the Catalogue Of Somatic Mutations In Cancer. Nucleic Acids Res. 2019;47: D941–D947.

35. Díaz-Gay M, Vangara R, Barnes M, Wang X, Islam SMA, Vermes I, et al. Assigning mutational signatures to individual samples and individual somatic mutations with SigProfilerAssignment. Bioinformatics; 2023 Jul. Available: http://biorxiv.org/lookup/doi/10.1101/2023.07.10.548264

36. Rosenthal R, McGranahan N, Herrero J, Taylor BS, Swanton C. DeconstructSigs: delineating mutational processes in single tumors distinguishes DNA repair deficiencies and patterns of carcinoma evolution. Genome Biol. 2016;17: 31.

Supplementary Figures

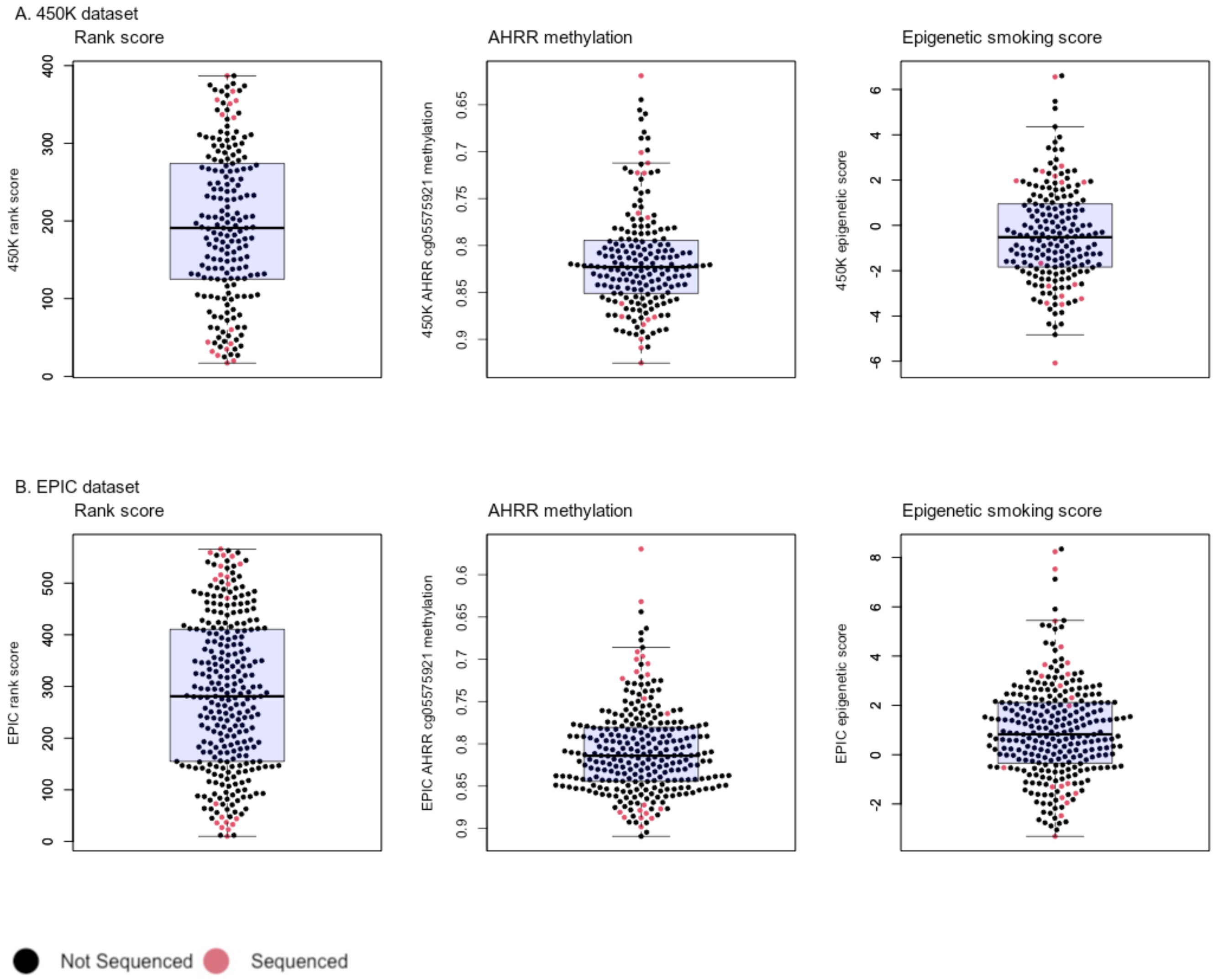

Figure S1. Distribution of combined rank score, AHRR methylation and epigenetic smoking score in sequenced vs non-sequenced subjects from the 450K and EPIC datasets. Genome-wide DNA methylation data were available from Illumina 450K methylation arrays and Illumina EPIC arrays. The overall ranked score was calculated by combining the ranks of the AHRR cg05575921 methylation biomarker with the ranks of the epigenetic score for each patient. Since AHRR CpG methylation level is inversely associated with prenatal tobacco exposure, we flipped the Y-axis (with descending order from low AHRR methylation level to high AHRR methylation level).

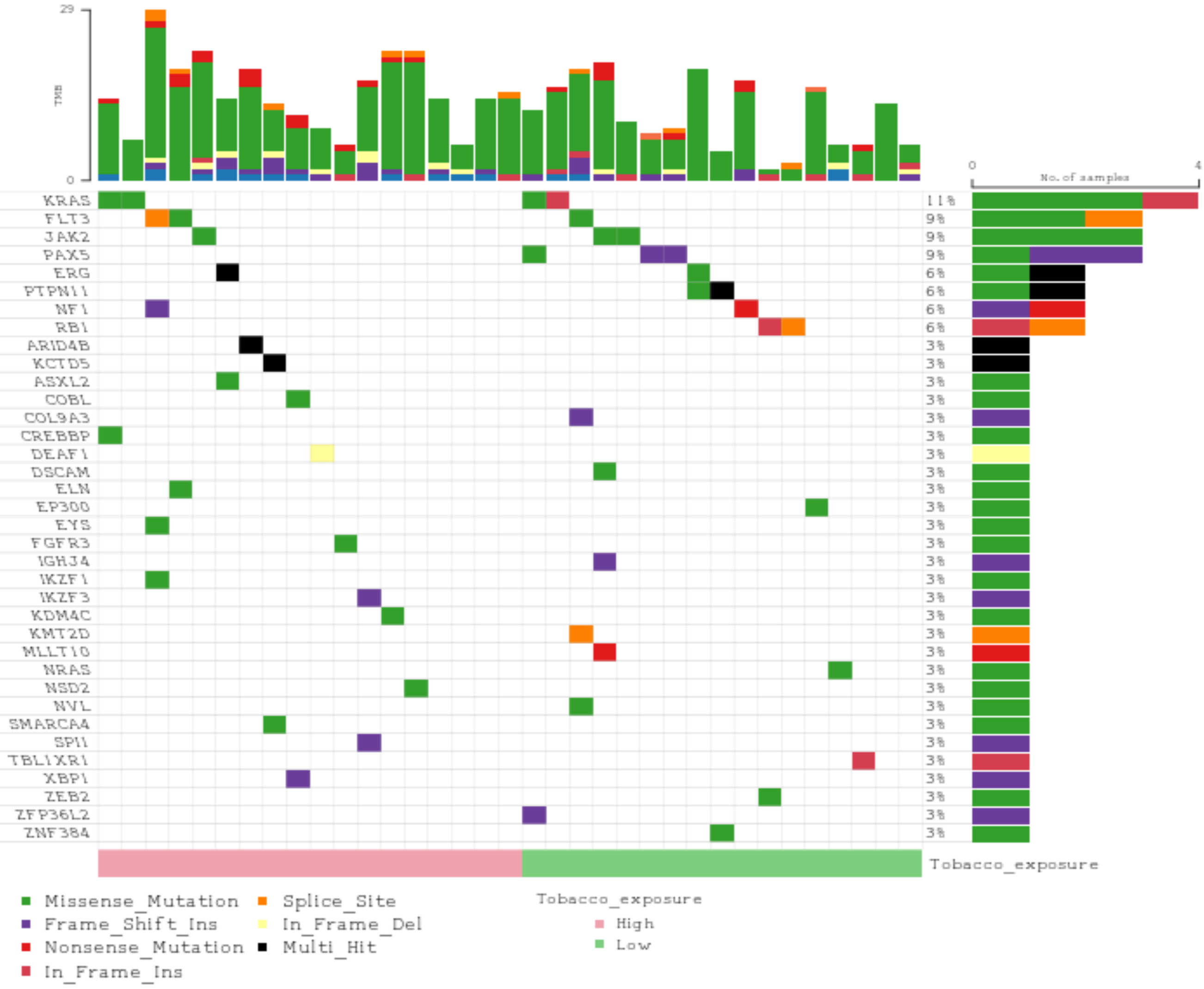

Figure S2. Oncoplot of somatic SNV/indels overlapping childhood ALL driver genes. Variants annotated as “Multi_Hit” are those genes which are mutated more than once in the same sample. “In_Frame_Ins”: in-frame insertion. “In_Frame_Del”: in-frame deletion. ”Frame_Shift_Ins”: frame-shift insertion. The right bar shows the number of samples with driver gene alteration. The top bar shows the tumor mutation burden in each sample.

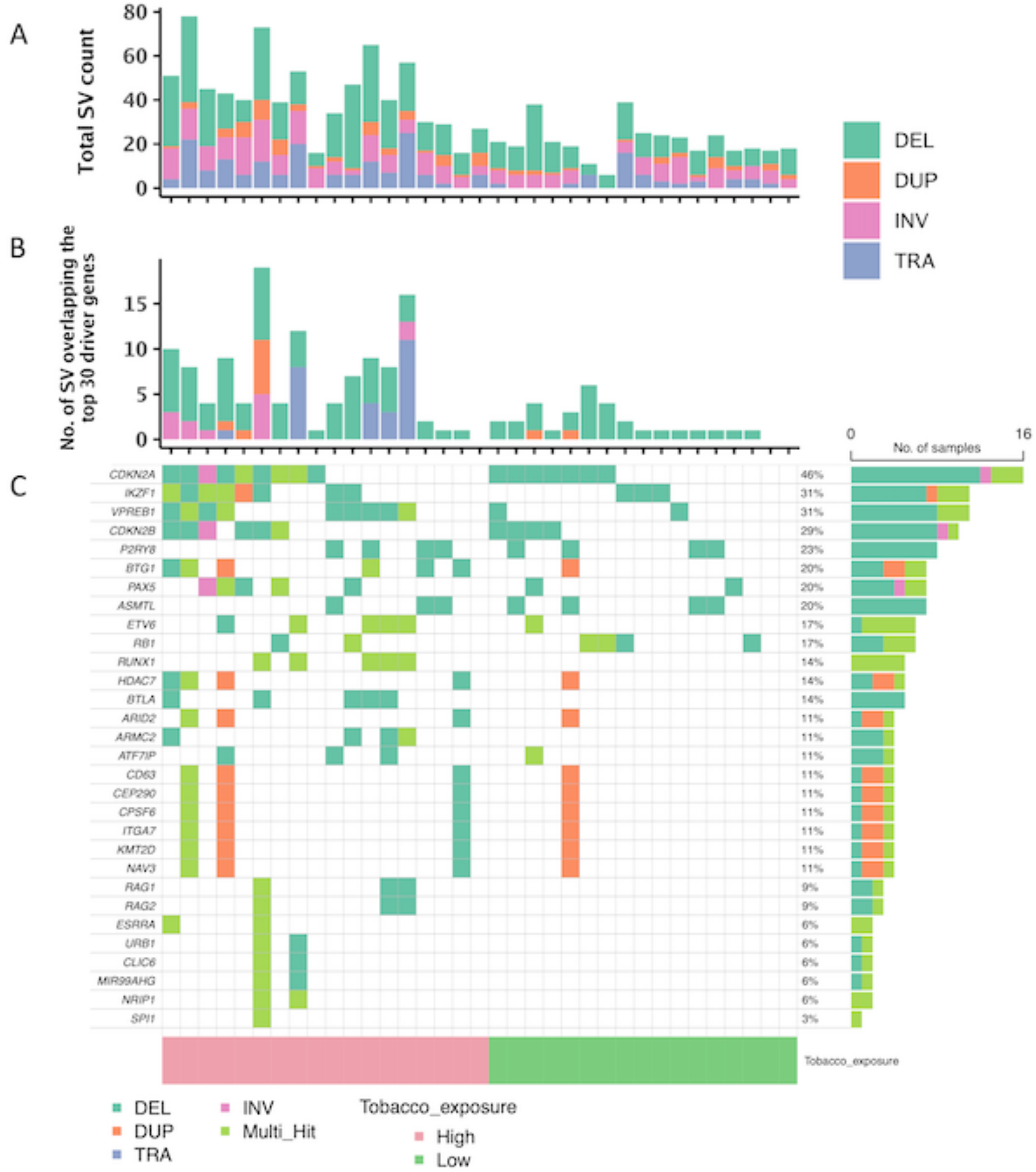

Figure S3 Somatic structural variants and their overlap with childhood ALL driver genes. The total number of structural variants (SVs) per patient (**A**) was based on the overlap of calls by three SV detection tools (Lumpy, Manta, and Delly), with final numbers of SVs based on those detected by at least two out of three methods. Total SV count was based on the sum of counts of different SV types: deletions (DEL), duplications (DUP), translocations (TRA), and inversions (INV). The middle plot (**B**) displays the number of SVs and different SV types per patient that overlapped known childhood ALL driver genes, limited to the top 30 affected genes in our dataset. The bottom oncoplot (**C**) shows the ALL driver genes affected by SVs across the 35 patients, with 32 (91.4%) out of 35 patients harboring at least one SV overlapping an ALL driver gene, and genes ordered by the number of affected patients. SVs annotated as “Multi_Hit” indicate where the same gene was affected by more than one SV in the same patient. For all three plots, childhood ALL patients were stratified by tobacco exposure status as indicated.

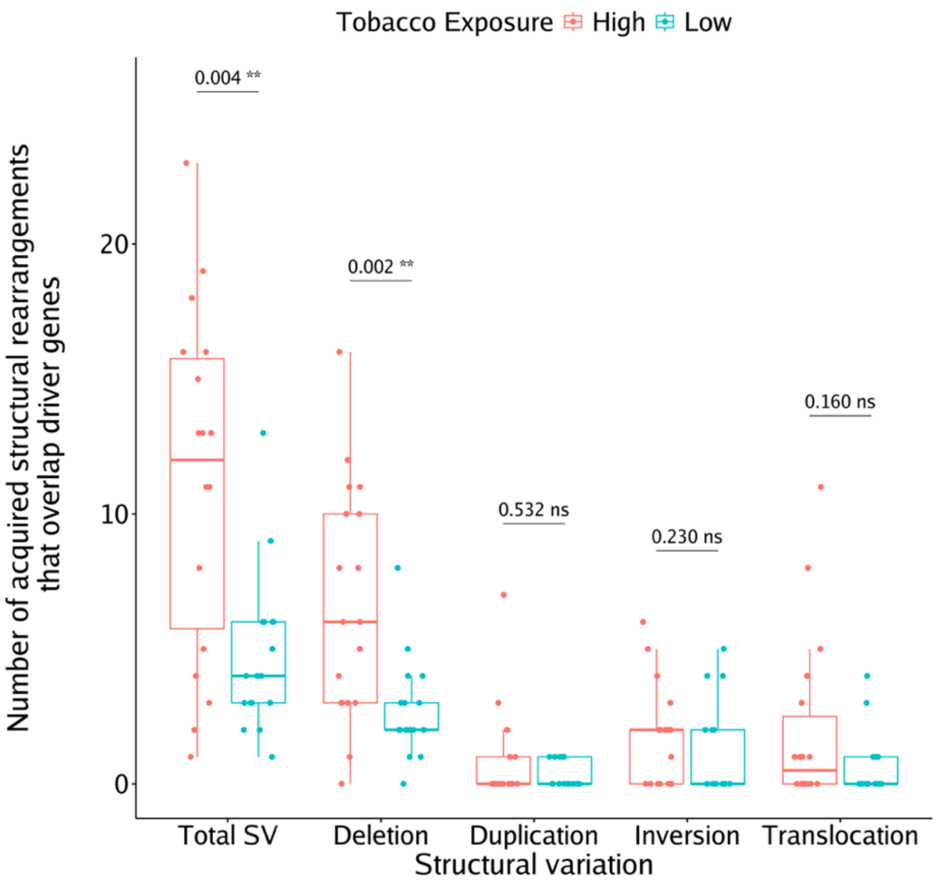

Figure S4. Number of genome-wide structural variants overlapping known ALL driver genes, by prenatal tobacco exposure in pediatric ALL.
Structural variants (SV) were all above 50 bases. Large structural rearrangements (deletions, duplications, inversions, and translocations) were called in 35 matched tumor/normal whole genome sequencing samples. We identified genome-wide somatic SVs that overlap known ALL driver genes. Box and whiskers plot of mutation per sample. *P* values from the Wilcoxon rank sum tests are shown in the figure. *** $P<.001$; ** $P<.01$; * $P<.05$

A. Full RAG12 heatmap (spacer: 12-bp) **B.** Full RAG23 heatmap (spacer: 23-bp)

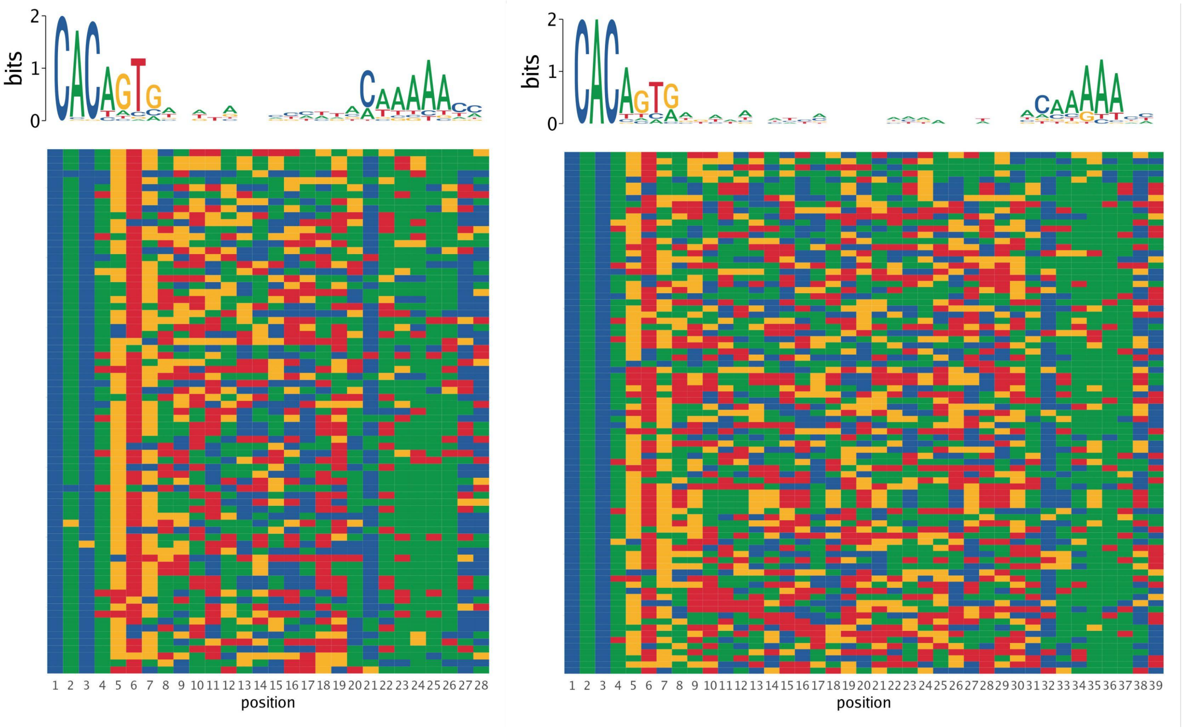

Figure S5. Heatmaps of full RSS motifs.
Each row in the heatmap represents a single deletion breakpoint sequence in our data. Each column represents a specific position. Cells are colored by the sequence at that position. Sequences are aggregated into a consensus logo aligned with the heatmap to visualize how sequence variability contributes to the motif. We plotted the occurrence pattern of non-Ig/TCR full RSS with 12 bp of intervening spacer (**A**) and the occurrence pattern of non-Ig/TCR full RSS with 23 bp of intervening spacer (**B**) within 50bp from the deletion breakpoints.

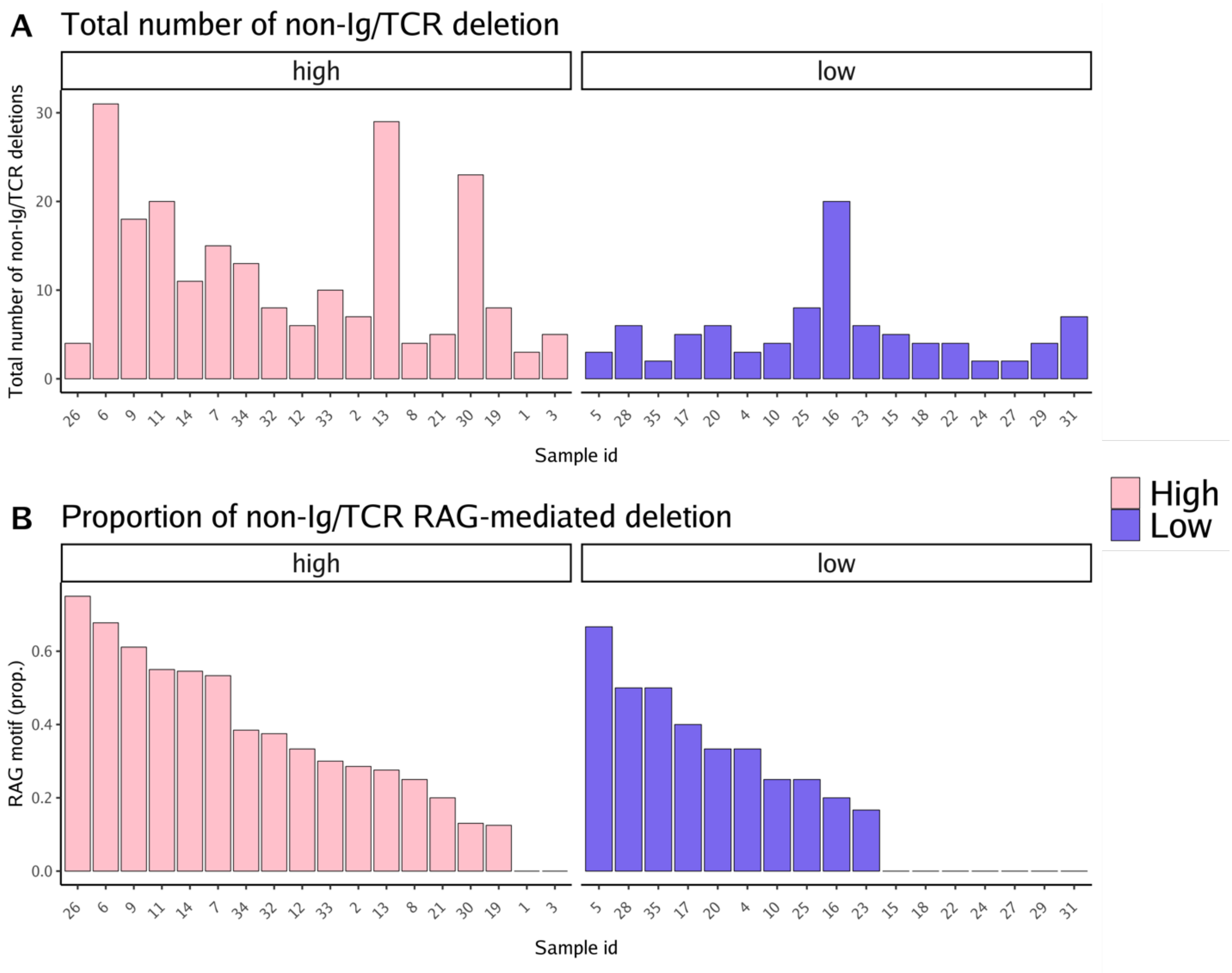

Figure S6. Total number of non-Ig/TCR deletions and proportion of putatively RAG-mediated non-Ig/TCR deletions, per patient and by tobacco exposure status.

Panel **A** shows the total number of non-Ig/TCR deletions per patient, in childhood ALL patients with high tobacco exposure (n=18) or low tobacco exposure (n=17). Panel **B** shows the proportion of non-Ig/TCR deletions that were putatively RAG-mediated (*i.e.*, deletions for which the RSS motif was detected by FIMO in at least one of the two breakpoints) per patient, in patients with high or low tobacco exposure. Patient IDs correspond to those included in Supplementary Table S2.

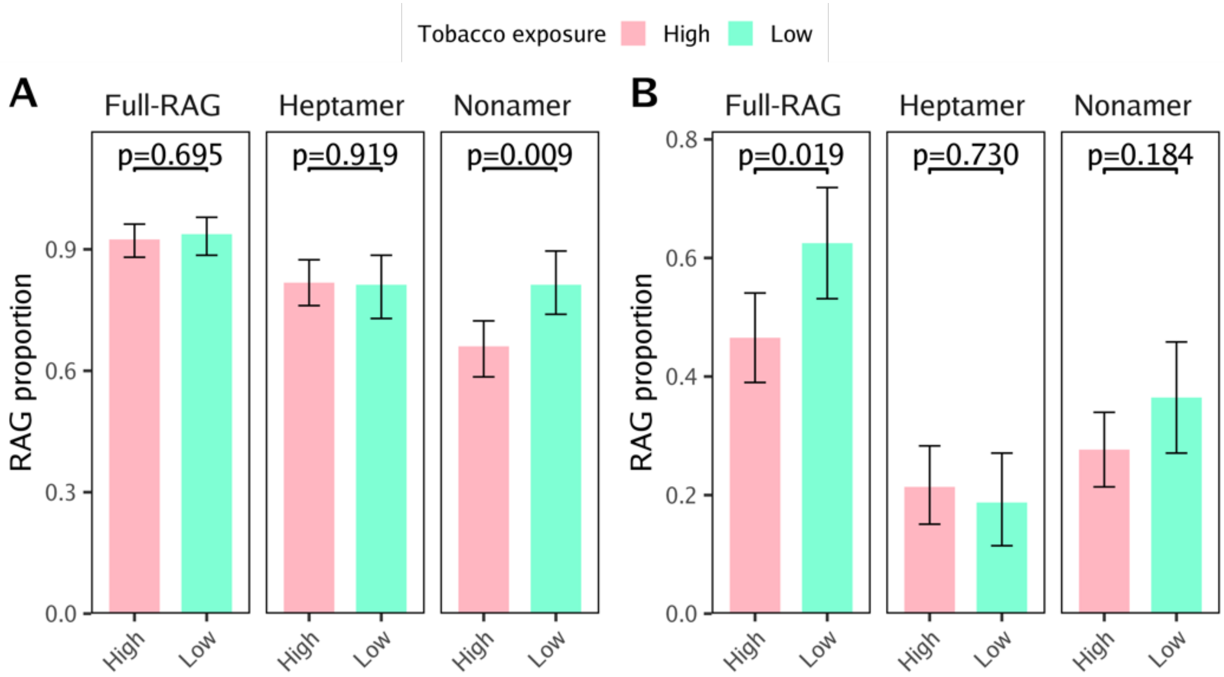

Figure S7. RAG-mediated deletions in the Ig/TCR regions by tobacco exposure status. (**A**) Proportion of deletions with at least one breakpoint at which an RSS motif was detected by FIMO. (**B**) Proportion of deletions with RSS motif detected at both breakpoints. Error bars represent 95% bootstrapped confidence intervals. Among 255 Ig/TCR deletions, 159 were from the high tobacco exposure group and 96 were from the low tobacco exposure group. *P* values from Chi-square tests for at least one RAG motif or Fisher's exact tests for RAG motif at both breakpoints are shown in the figure.

A

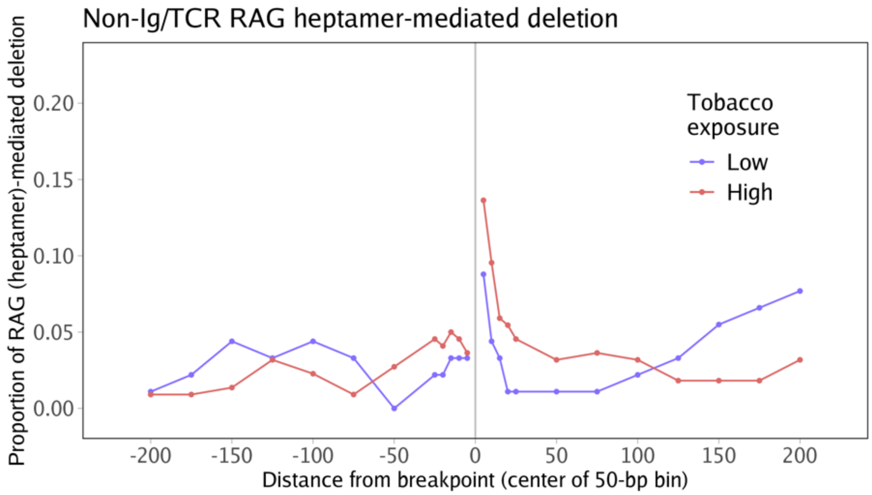

B

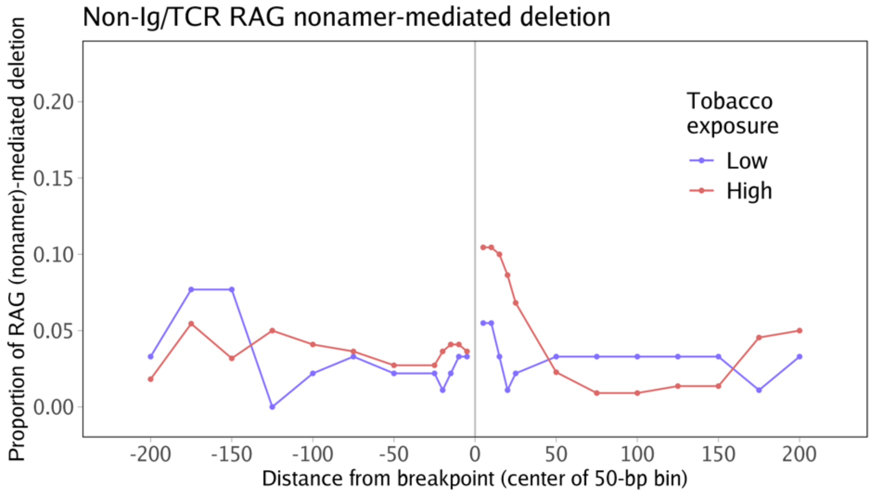

Figure S8. Proportion of non-Ig/TCR deletions with at least one RAG heptamer or nonamer motif as a function of distance from the breakpoint.

The proportion of off-target (non-Ig/TCR) RAG-mediated deletions with at least one RAG heptamer (**A**) or nonamer (**B**) motif was plotted against the distance of the motif from the deletion breakpoint, ranging from within 5-bp to 200-bp. A positive distance represents bases interior to the deletion breakpoint (inside the deletion) and a negative value represents bases exterior to the breakpoint (outside the deletion).

Rank	Motif	P-value	log P-pvalue	% of Targets	% of Background	STD(Bg STD)
1	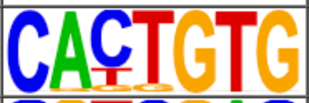	1e-35	-8.088e+01	14.42%	3.75%	15.9bp (15.2bp)
2 *	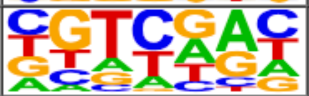	1e-10	-2.322e+01	3.73%	0.90%	9.1bp (13.0bp)
3 *	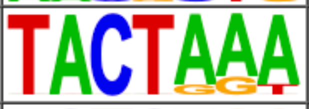	1e-9	-2.153e+01	4.57%	1.39%	12.2bp (12.5bp)
4 *	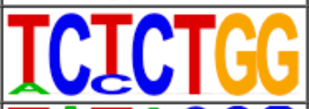	1e-9	-2.149e+01	6.61%	2.57%	12.6bp (12.6bp)
5 *	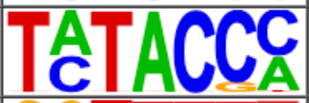	1e-8	-1.984e+01	6.85%	2.85%	12.7bp (13.5bp)
6 *	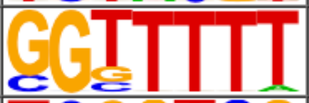	1e-8	-1.971e+01	7.81%	3.49%	10.1bp (13.1bp)
7 *	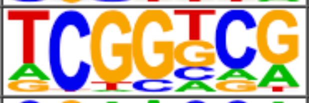	1e-8	-1.943e+01	1.68%	0.21%	12.5bp (12.0bp)
8 *	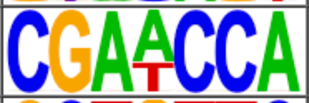	1e-8	-1.853e+01	1.44%	0.15%	10.2bp (12.7bp)
9 *	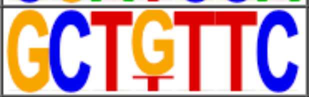	1e-7	-1.731e+01	4.45%	1.57%	9.7bp (14.0bp)
10 *	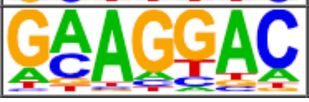	1e-6	-1.451e+01	21.51%	15.09%	11.6bp (12.4bp)

Figure S9. Off-target RAG-mediated deletions identified by HOMER *de novo* motif scanning with lengths of 7bp.

This table displays results from HOMER *de novo* motif discovery analysis for motifs with length=7bp found in sequences +/- 50 bp from each deletion breakpoint, limited to non-Ig/TCR (off-target) deletions. The top 5 most significant motifs are displayed. The most significant motif "CACTGTG" corresponds to the RAG heptamer. Motifs labeled with asterisks were possible false positive results according to HOMER.

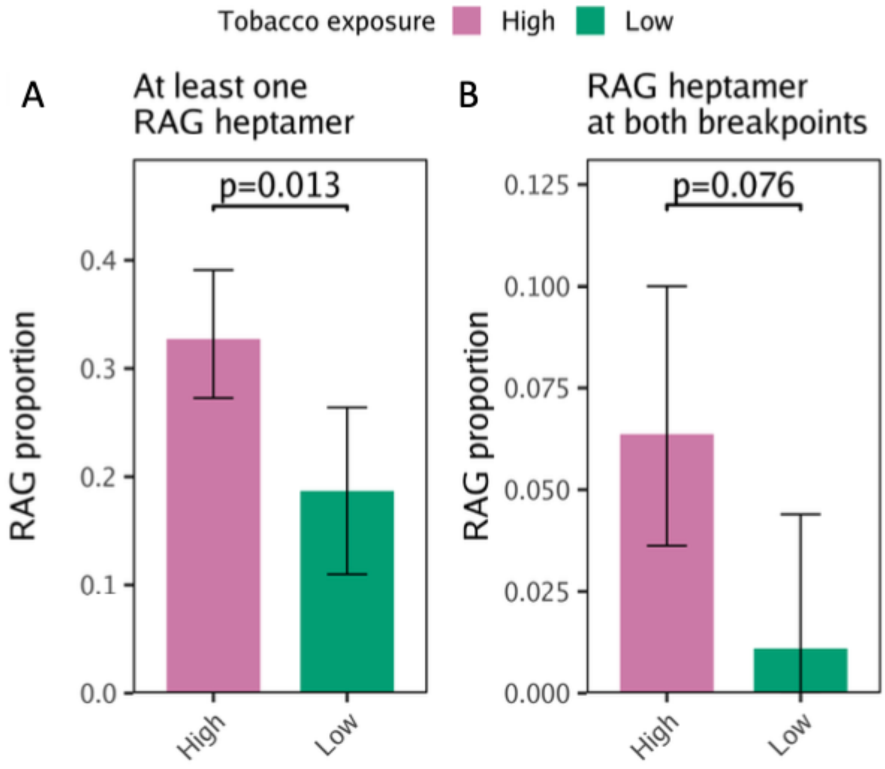

Figure S10. Proportion of non-Ig/TCR deletions with at least one RAG heptamer or RAG heptamer at both breakpoints identified by HOMER *de novo* motif scanning with lengths of 7bp.
Bar plots below show the proportion of deletions with at least one breakpoint (*p* value from Chi-square test) (**A**) or with both breakpoints (*p* value from Fisher's exact test) (**B**) at which the RAG heptamer was identified by HOMER, in childhood ALL patients with high tobacco exposure (n=18) or low tobacco exposure (n=17).

Rank	Motif	P-value	log P-pvalue	% of Targets	% of Background	STD(Bg STD)
1	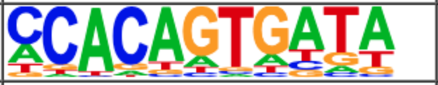	1e-45	-1.039e+02	8.89%	0.97%	15.1bp (14.4bp)
2	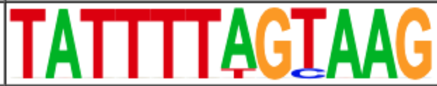	1e-31	-7.237e+01	1.68%	0.01%	7.0bp (10.3bp)
3	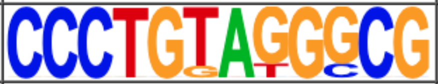	1e-29	-6.872e+01	1.44%	0.00%	5.8bp (4.1bp)
4	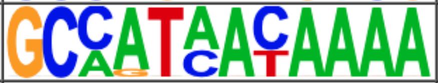	1e-24	-5.738e+01	1.56%	0.01%	5.8bp (9.2bp)
5	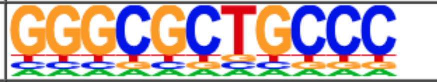	1e-24	-5.570e+01	1.20%	0.00%	3.5bp (6.0bp)
6	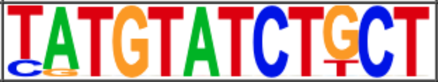	1e-24	-5.570e+01	1.20%	0.00%	16.1bp (0.0bp)
7	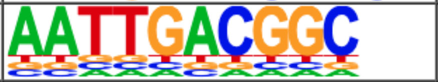	1e-21	-4.934e+01	1.08%	0.00%	9.2bp (0.0bp)
8	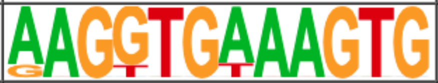	1e-21	-4.934e+01	1.08%	0.00%	7.4bp (0.0bp)
9	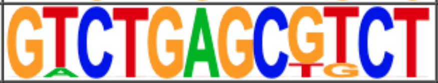	1e-21	-4.934e+01	1.08%	0.00%	3.6bp (0.0bp)
10	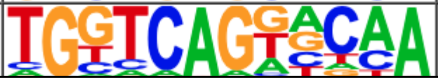	1e-21	-4.865e+01	1.68%	0.02%	11.2bp (6.5bp)

Figure S11. Off-target RAG-mediated deletions identified by HOMER *de novo* motif scanning with lengths of 5 to 12bp.
The size of region for motif finding equals +/- 50 bp from the breakpoint. Motif length is equal to 5-12 bp. The most significant motif, which includes "CACAGTG", corresponds to the RAG heptamer, and was the only motif found in >2% of targets.

A. SBS signatures selection plot

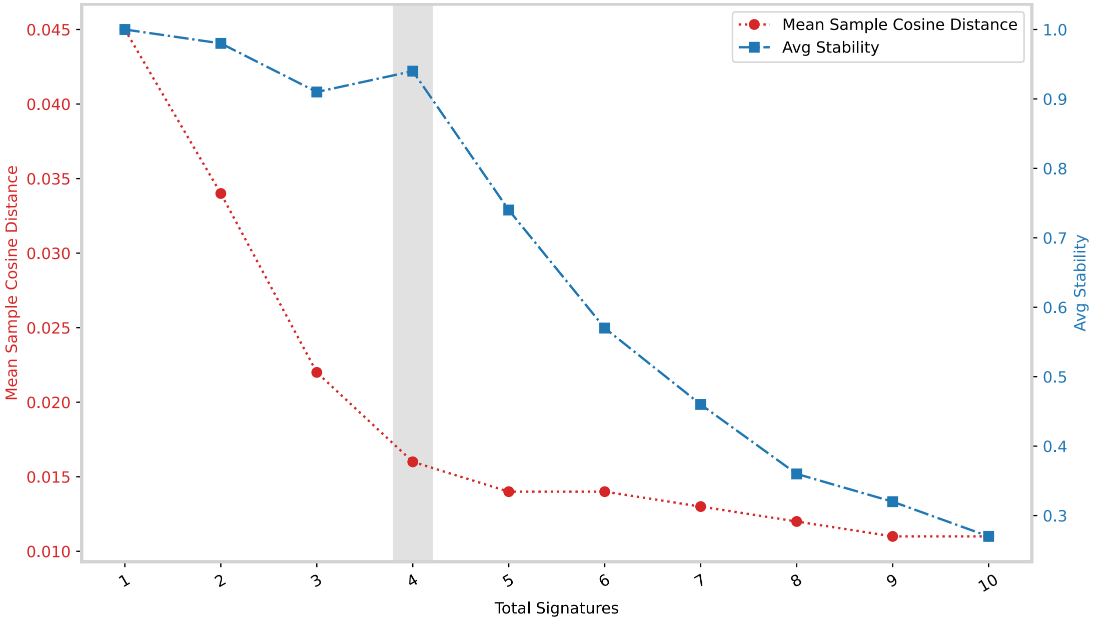

.

B. Number of mutations in each de novo mutational signature from SigProfilerExtractor

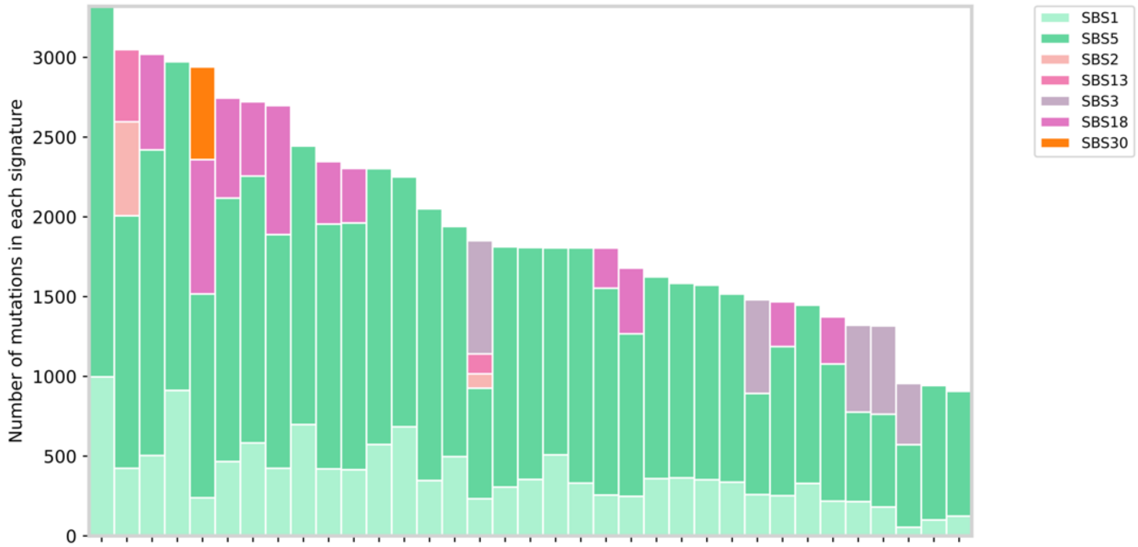

Figure S12. De novo mutational signature results (overall).
A: Four de novo SBS signatures were considered as the best solution with the highest stability (average Silhouette score=0.94) and relative low mean sample Cosine distance (0.016).
B: *De novo* extraction analysis using SigProfilerExtractor. Four de novo SBS were decomposed into seven COSMIC signatures. SBS1 and SBS5: clock-like signature. SBS2 and SBS13: AID/APOBEC family of cytidine deaminases. SBS3: Defective homologous recombination-based DNA damage repair. SBS18: Possibly damage by reactive oxygen species. SBS30: Deficiency in base excision repair due to inactivating mutations in *NTHL1*.

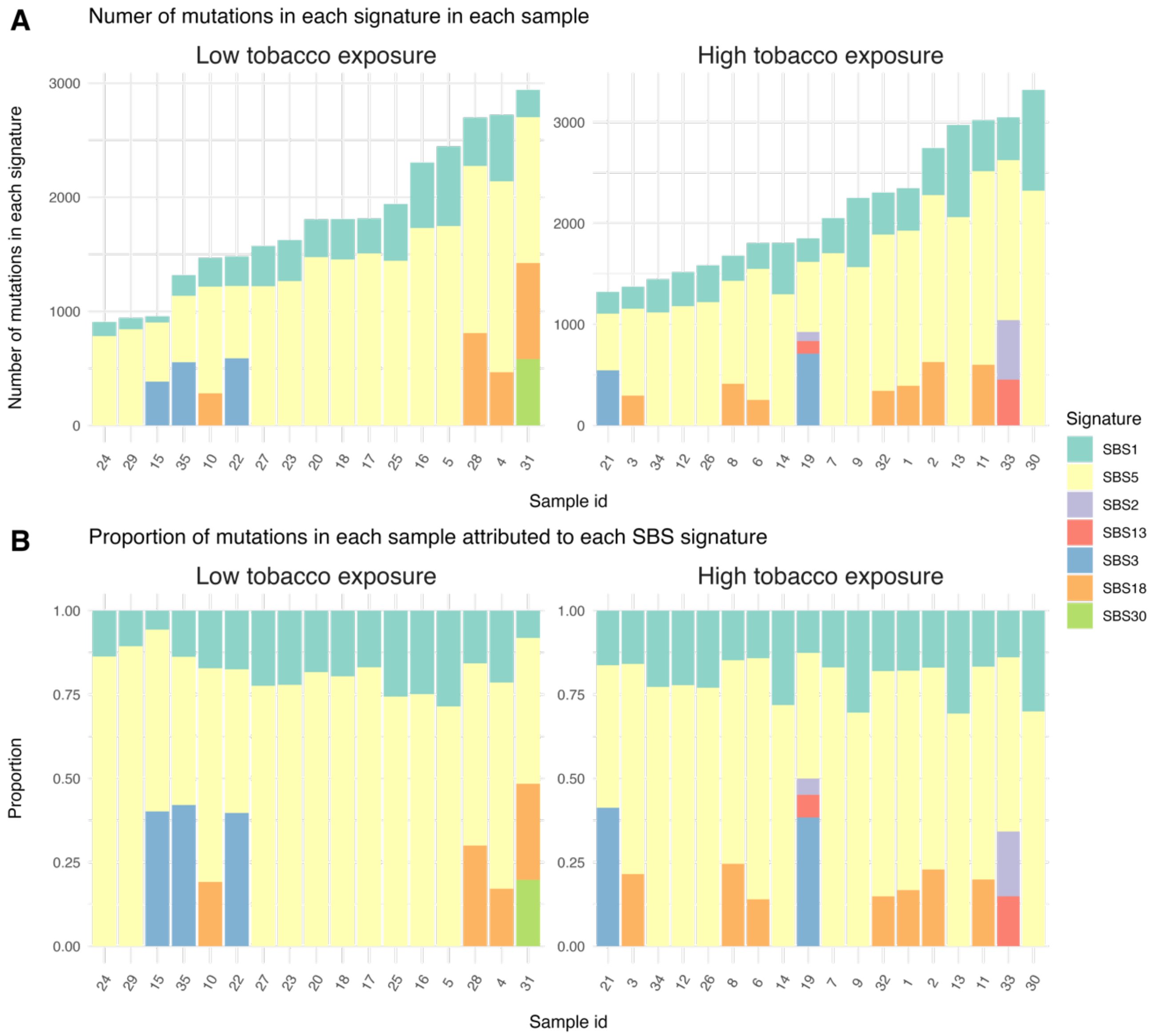

Figure S13. Number and proportion of *de novo* mutational signatures by tobacco exposure and patient based on SigProfilerExtractor result.

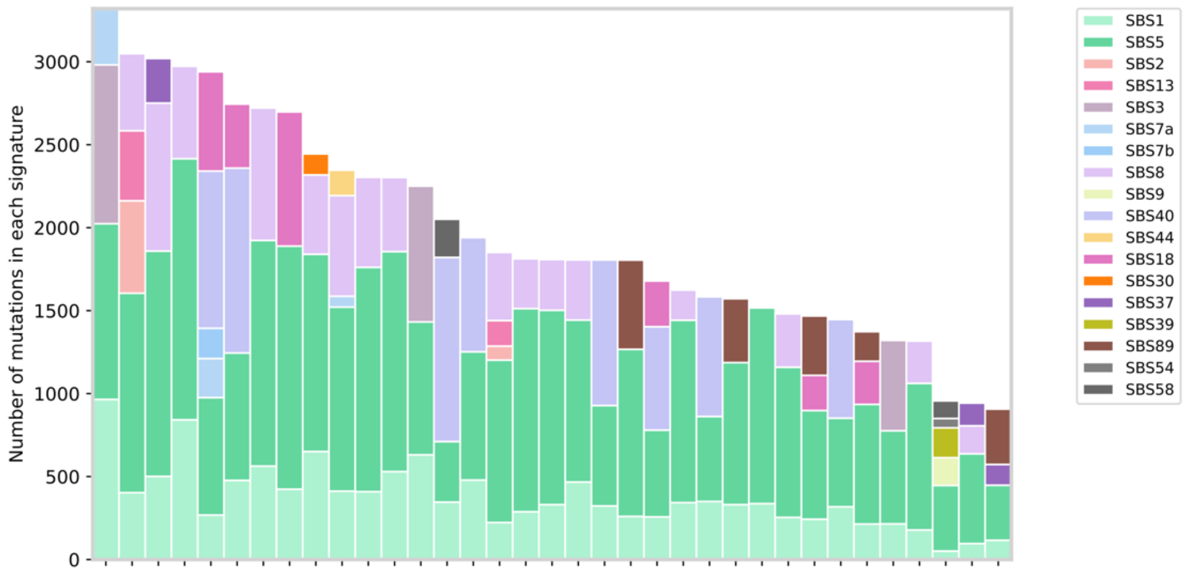

Figure S14. Fitting previously known mutational signatures to each patient. Assigning previously known mutational signatures to individual patients and individual somatic mutations. A total of 18 signatures were identified. SBS 1, SBS5: clock-like in that the number of mutations in most cancers and normal cells correlates with the age of the individual; SBS2, SBS13: AID/APOBEC family of cytidine deaminases; SBS3: defective homologous recombination-based DNA damage repair; SBS7a, SBS7b: ultraviolet light exposure; SBS8, SBS37, SBS39, SBS40, SBS89: unknown; SBS 9: Polymerase eta somatic hypermutation activity; SBS44: Defective DNA mismatch repair; SBS18: Possibly damage by reactive oxygen species. SBS30: Deficiency in base excision repair due to inactivating mutations in *NTHL1*; SBS54, SBS58: possible artefact.

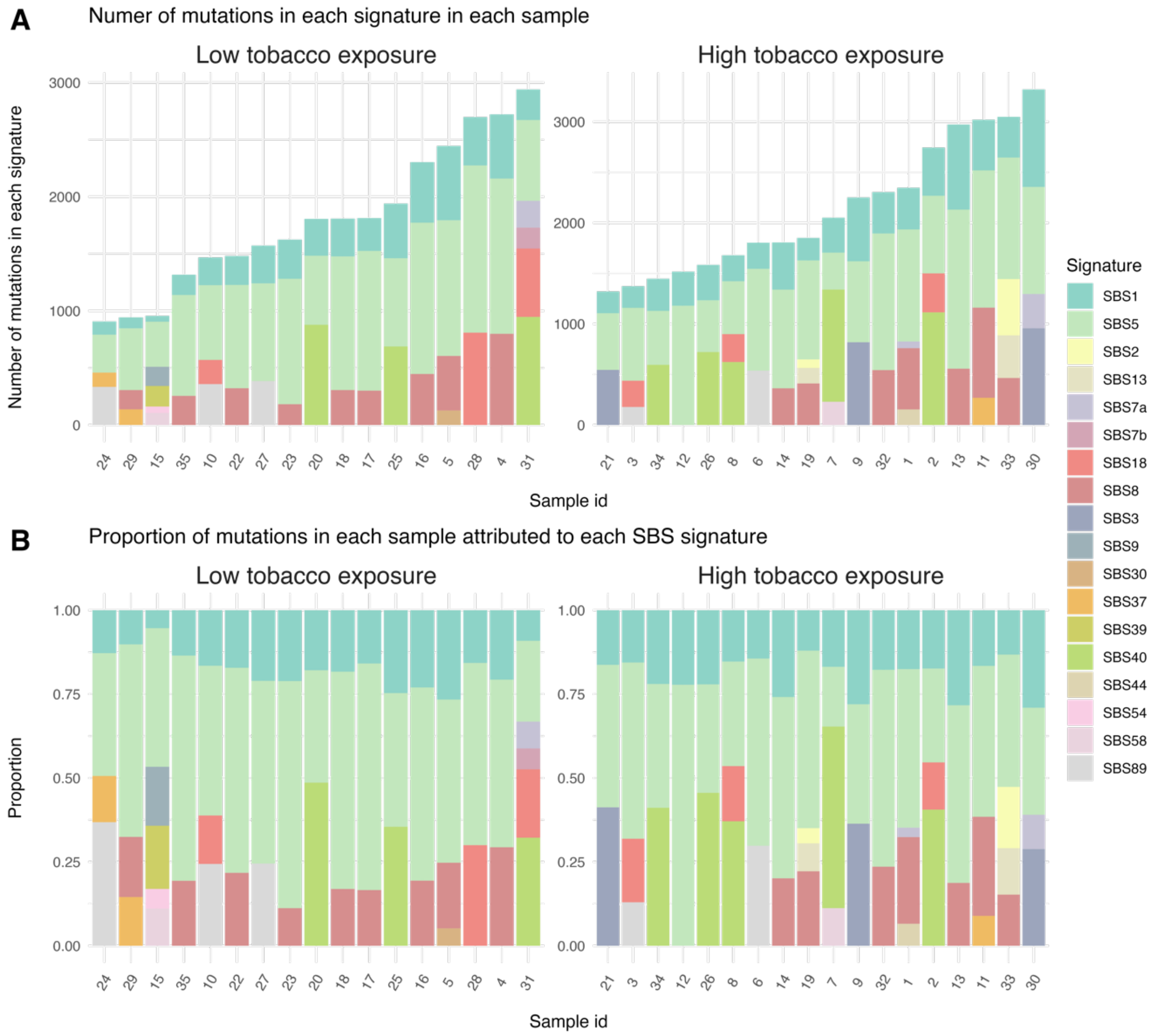

Figure S15. Number and proportion of previously known mutational signatures by tobacco exposure and patient based on SigProfilerAssignment result.

Proportion of known mutational signatures in the overall patients

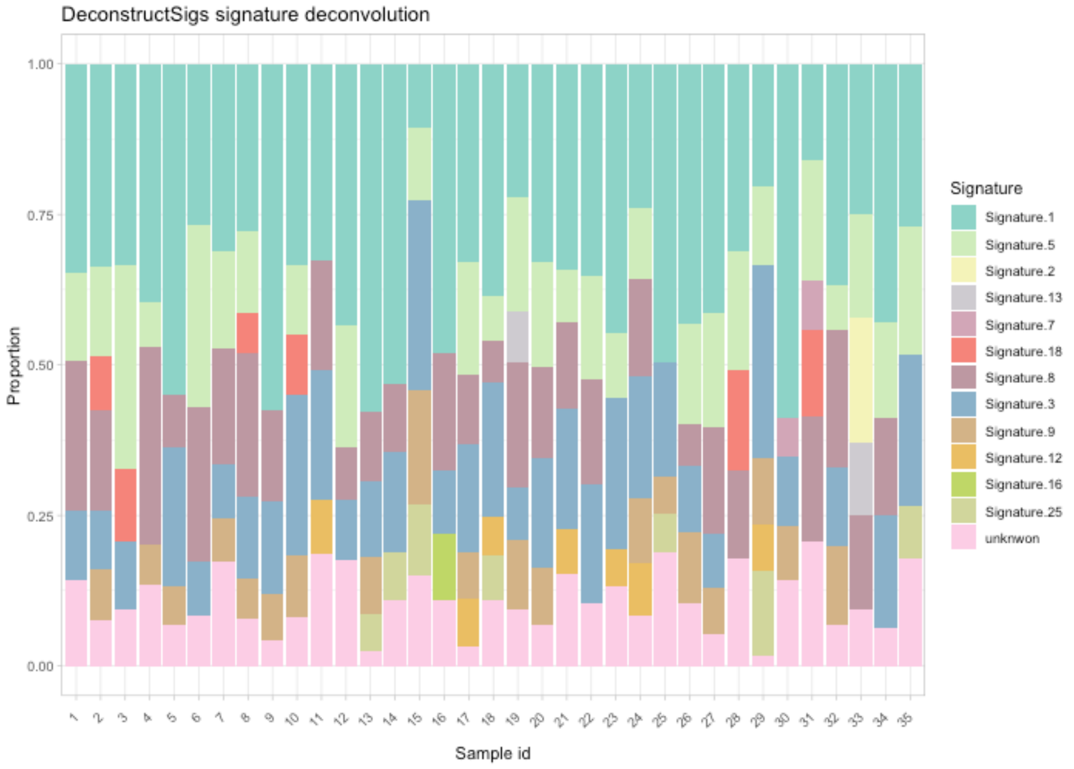

Proportion of known mutational signatures by tobacco exposure and patient

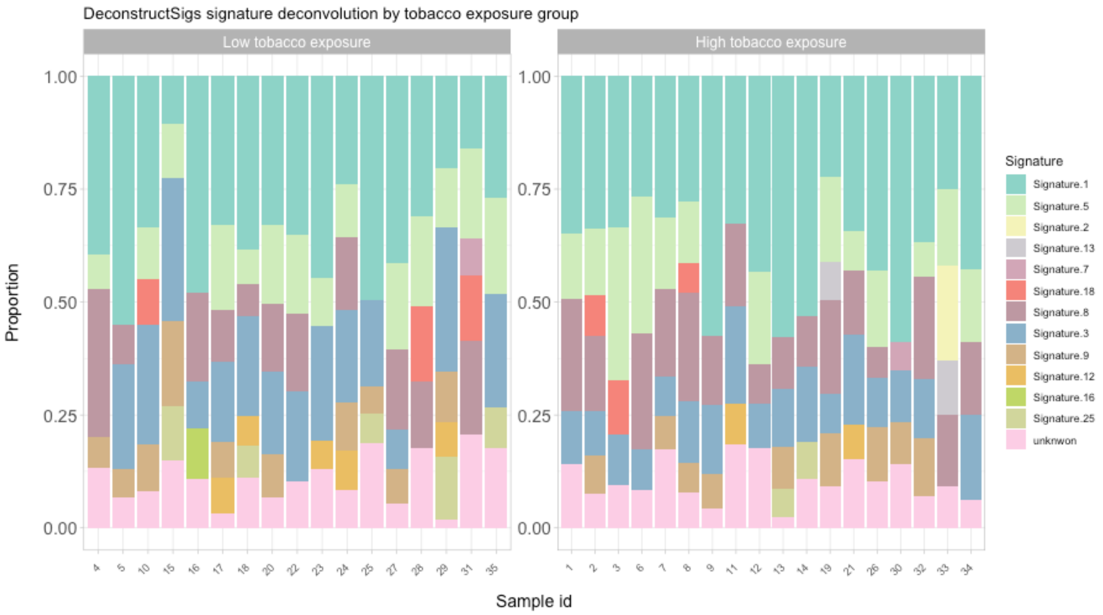

Figure S16. Fitting known mutational signature using DeconstructSigs.

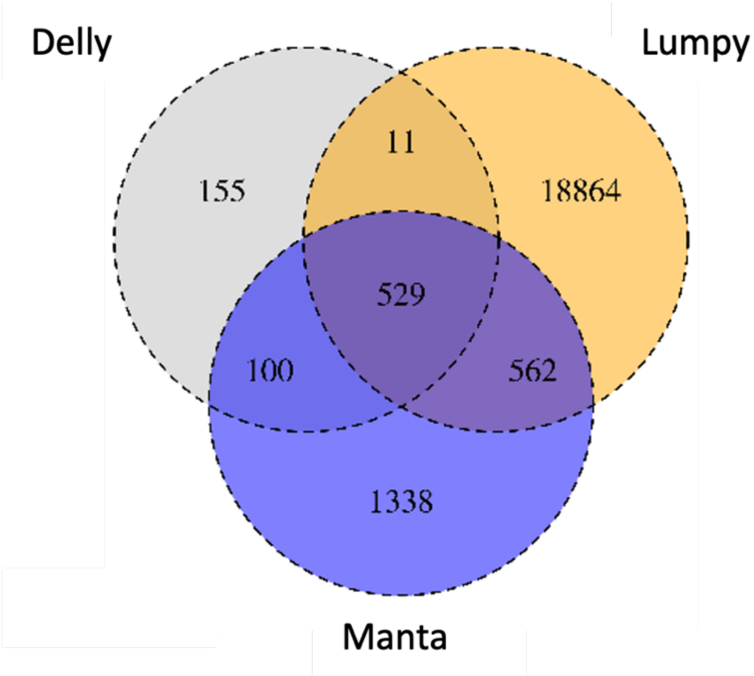

Figure S17. Venn diagram displaying the number of structural variants called by Lumpy, Manta and Delly.

There were 19,964 SVs, 2,529 SVs, 795 SVs called by Lumpy, Manta and Delly, respectively. A total of 1202 SVs identified by at least two callers, including 600 deletions, 103 duplications, 287 inversions and 212 translocations. After excluding VAF < 0.1, the final dataset for downstream analysis included 566 deletions, 90 duplications, 273 inversions and 211 translocations, yielding a total of 1140 structural variants.
